# Daily biomarker trajectories predict major bleeding in patients on venovenous ECMO for ARDS: a retrospective longitudinal cohort study

**DOI:** 10.1038/s41598-026-47560-0

**Published:** 2026-04-09

**Authors:** Thomas Stueber, Jil-Madeline Homeier, Hans-Joerg Gillmann, Jona Wassong, Carolin Jung

**Affiliations:** Department of Anesthesiology and Intensive Care Medicine, Hannover Medical School, Carl-Neuberg-Strasse 1, 30625 Hannover, Germany

**Keywords:** ARDS, ECMO, Coagulation, Bleeding, Circuit exchange, Biomarkers, Diseases, Medical research, Risk factors

## Abstract

Bleeding complications during venovenous extracorporeal membrane oxygenation (ECMO) remain a leading cause of morbidity, yet granular longitudinal data linking daily biomarker dynamics to bleeding around circuit exchanges are scarce. We analyzed 93 exchanges from 35 severe acute respiratory distress syndrome (ARDS) patients (2020–2022) across 15-day peri-exchange windows in this retrospective single-center cohort study. Major bleeding (defined by ELSO criteria) occurred on 100/1317 observation-days (7.6%), clustering peri-exchange (13.1% vs. 4.2% outside ± 2 days, *p* < 0.001). Using generalized linear mixed models (GLMMs) with nested random effects, we characterized coagulation trajectories and bleeding predictors. White blood cell count was the strongest predictor (OR 2.00/SD, 95% CI 1.43–2.82, *p* < 0.001), followed by platelets (OR 0.58, *p* = 0.014) and fibrinogen (OR 0.60, *p* = 0.035). Time-lagged GLMMs identified D-dimer and fibrinogen as early warning markers 1–2 days pre-bleeding. Circuit exchange shortened bleeding duration (HR 2.23, 95% CI 1.16–4.29, *p* = 0.017). D-dimer rose pre-exchange and declined post-exchange, while fibrinogen and platelets showed inverse patterns, suggesting consumptive coagulopathy reversed by exchange. White blood cell trajectories implicated independent inflammatory risk. These findings demonstrate a 1–2 days predictive time window for any action needed to counteract or prevent major bleeding, supporting biomarker-guided circuit management and hemostatic strategies in ECMO.

**Supplementary Information:**

The online version contains supplementary material available at 10.1038/s41598-026-47560-0.

## Introduction

In patients with severe ARDS refractory to conventional ventilation, venovenous extracorporeal membrane oxygenation (VV-ECMO) provides lifesaving support by enabling extracorporeal gas exchange and allowing lung-protective ventilation^1^. Despite technological advances and improved anticoagulation strategies, bleeding complications remain a leading cause of morbidity and mortality in ECMO patients^2,3^, driven by complex interactions of hemostatic consumption, anticoagulation, and circuit-related activation of coagulation and fibrinolysis^4^. The significance of D-dimer as a predictor of bleeding and circuit thrombosis in this context remains debated, with some studies linking rising D-dimer to oxygenator thrombus formation and consumptive coagulopathy^5–10^, while others argue that elevated D-dimer levels may reflect impaired coagulation without necessarily justifying specific interventions^11–13^. As coagulation and bleeding risk are inherently dynamic processes^2,14^, granular longitudinal data are needed for a more comprehensive assessment^15^. ECMO circuit exchange may attenuate consumptive coagulopathy by removing the thrombotic burden within the circuit, but its effects on coagulation dynamics and the optimal timing of circuit exchanges warrant further investigation^14^. In this study, we analyzed daily trajectories of coagulation, inflammation, and ECMO circuit degradation markers before and after ECMO circuit exchange in patients with COVID-19 ARDS, systematically recorded major bleeding events, and sought to identify their risk factors. We aimed to characterize the relationship between circuit-related consumptive coagulopathy, systemic inflammation, and bleeding risk to inform future strategies for individualized hemostatic management.

## Methods

### Study design

This retrospective single-center cohort study was conducted at Hannover Medical School’s mixed ICU (Germany, 2020–2022). The institutional ethics committee approved the study (No. 11276_BO_SK_2024, February 29, 2024) with waiver of informed consent. The study adheres to STROBE guidelines^16^ and was registered (DRKS00038439) with pre-specified statistical analysis plan. The pre‑specified plan included machine‑learning models and formal discrimination metrics, but these were not pursued because the number of bleeding events was insufficient for reliable training and validation.

### Patient population

We enrolled adults (≥ 18 years) with severe COVID-19 ARDS requiring VV-ECMO who underwent ≥ 1 circuit exchange. From 35 patients, we analyzed 93 exchanges across 15-day observation windows centered on each exchange (day 0 = exchange day, observation from day − 7 to day + 7). Patients contributed 1–10 exchanges (median: 2); each exchange was treated as an independent case, with within-patient correlation accounted for via random effects. An additional 18 patients with severe COVID-19 ARDS managed on VV-ECMO who did not undergo circuit exchange during the study period were analyzed descriptively for context. Patients received therapeutic anticoagulation with unfractionated heparin (target aPTT 50 s or anti-Xa 0.4–0.6 IU/mL) or argatroban (target anti-IIa 0.5–1.0 µg/mL) in cases of heparin-induced thrombocytopenia or suspected heparin resistance.

### Definitions and collected variables

The primary outcome was major bleeding, defined per ELSO criteria as clinically overt bleeding with hemoglobin drop ≥ 2 g/dL, bleeding volume > 20 mL/kg, transfusion > 10 mL/kg packed red blood cells/24 h, or bleeding requiring surgical intervention, including retroperitoneal, pulmonary, or central nervous system hemorrhage^17^. Bleeding events were retrospectively identified from medical records and classified as binary daily outcomes. ARDS was defined per Berlin criteria. Complete ECMO circuit exchange (oxygenator, pump, and circuit tubing) was modeled as a time-varying intervention on Day 0, with sequential exchange number as covariate to account for cumulative exposure. Data collected included demographics, clinical outcomes, daily laboratory parameters (coagulation, hemolysis, inflammatory markers), ECMO performance indicators, and transfusion requirements. D-dimer values were measured using the Innovance D-dimer assay (Siemens Healthineers, Marburg, Germany) and censored at the upper detection limit of 35.2 mg/L.

### Data management and handling of missing data

Details on data structure and preprocessing are provided in Supplementary Methods. Briefly, time points after death, ECMO decannulation, or before ICU admission were excluded as structurally missing. We applied multiple imputation by chained equations (MICE) with predictive mean matching across 30 imputed datasets, pooled using Rubin’s rules. Convergence and distributional validity were confirmed (Supplementary Methods, Fig. S2). Sensitivity analyses compared multiply imputed with complete-case results (Fig. S3). Multicollinearity among laboratory predictors was assessed across all imputed datasets (Fig. S1).

### Statistical analysis

#### Longitudinal trajectories

Coagulation, hematologic, and hemolysis parameters were analyzed using linear mixed models (LMMs) with nested random intercepts for episodes within patients to account for repeated measures and the hierarchical data structure. Analyses spanned 15-day windows centered on circuit exchange (day 0). Fixed effects included linear and quadratic time terms, intervention phase (pre vs. post-exchange), a time-by-phase interaction to capture trajectory changes following exchange, major bleeding occurrence, and sequential exchange number.

#### Risk factors for major bleeding

Generalized linear mixed models (GLMM) with a binomial distribution and logit link function were used to assess predictors of major bleeding within the observation period. Models included nested random intercepts for episodes within patients to account for repeated measurements and the hierarchical data structure. Time was modeled using natural cubic splines with 2 degrees of freedom centered at the exchange day to allow for non-linear temporal trends. Laboratory predictors (fibrinogen, platelet count, D-dimer, INR, free hemoglobin, white blood cell count, post-oxygenator PO_2_) were selected a priori based on pathophysiological relevance and z-standardized to enable comparison of effect sizes. Odds ratios represent the change in bleeding risk per 1 standard deviation (SD) increase. Models were fitted across 30 imputed datasets and pooled using Rubin’s rules. Fraction of missing information (FMI) was calculated to quantify the impact of missingness on parameter estimates.

#### Exploratory analyses

To assess temporal relationships between biomarkers and subsequent bleeding, we fitted multivariable GLMMs including D-dimer, fibrinogen, platelet count, and white blood cell count simultaneously as z-standardized lagged predictors at each lag interval (1–7 days). Predictor selection was based on the primary multivariable model. aPTT was excluded due to confounding by therapeutic anticoagulation. Complementary univariate lagged analyses are reported in Table S8. In post-hoc analyses, daily bleeding status was modeled as a two-state Markov process using a continuous-time multi-state model (msm package) to estimate transition intensities, mean sojourn times, and hazard ratios for circuit exchange. As msm does not accommodate clustered observations, GLMMs with nested random intercepts were fitted as sensitivity analyses (Supplementary Methods). Additional descriptive analyses characterized trajectories in patients without circuit exchange and during the terminal phase. All analyses were performed in R (version 4.5.1) using the mice, msm, lme4, and lmerTest packages. Statistical significance was set at *p* < 0.05 (two-sided).

## Results

### Study population and bleeding incidence

**Fig. 1:**
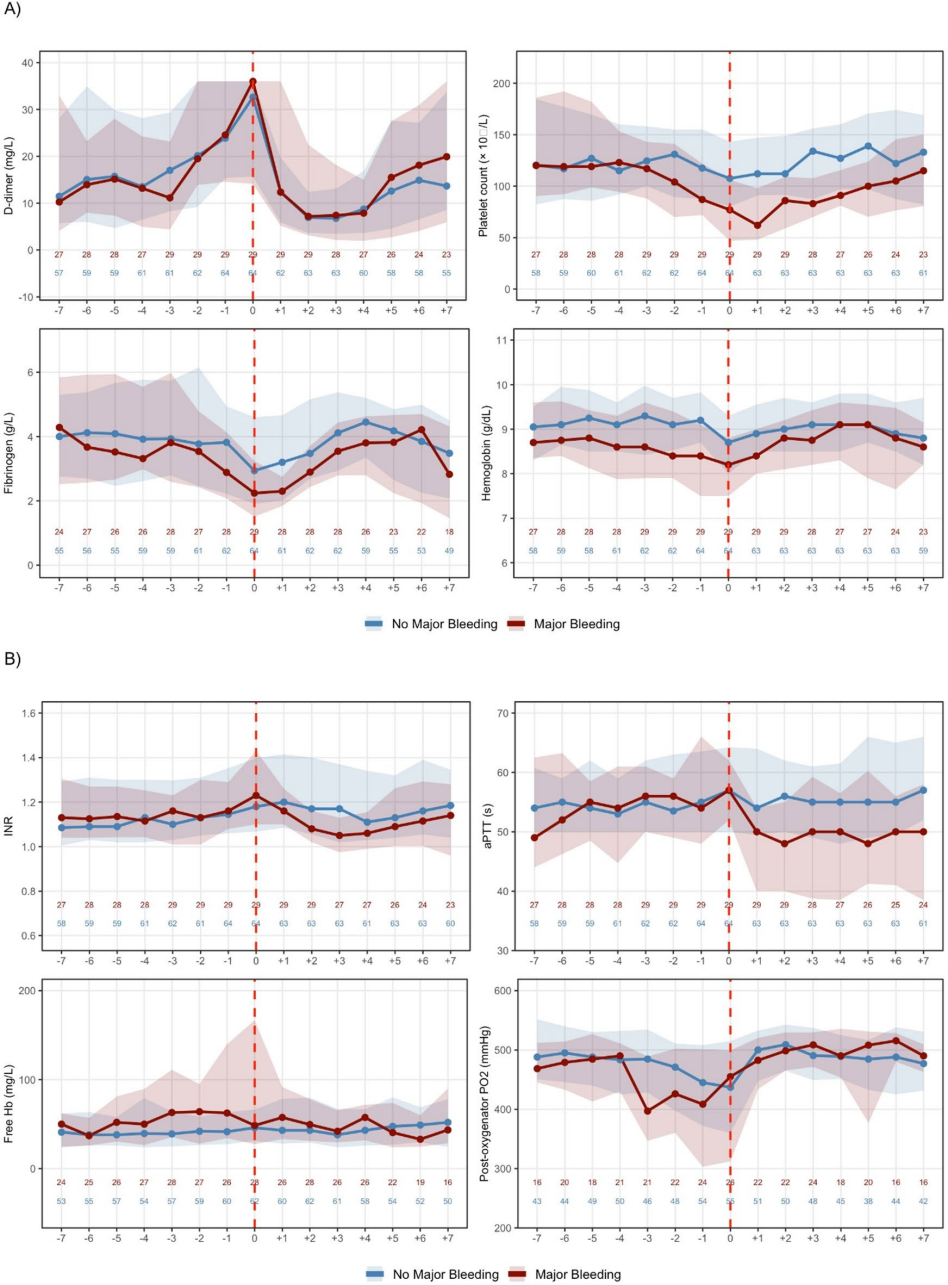
Laboratory parameter trajectories around ECMO circuit exchange, stratified by major bleeding status. (**A**) D-dimer, platelet count, fibrinogen, and hemoglobin trajectories. D-dimer values are censored at the upper detection limit of 35.2 mg/L. (**B**) INR, aPTT, free hemoglobin, and post-oxygenator PO_2_ trajectories. Data from 93 ECMO circuit exchange episodes in 35 patients, stratified by occurrence of major bleeding during the 15-day observation period (red, *n* = 29) versus no major bleeding (blue, *n* = 64). Lines represent medians; shaded areas, interquartile range. Day 0 (dashed line) indicates circuit exchange. Numbers denote sample size at each timepoint. Cases with major bleeding showed lower platelet counts and fibrinogen levels, with a nadir around the day of exchange, whereas D-dimer levels peaked at exchange. Both groups demonstrated partial recovery of coagulation parameters following circuit exchange. *Abbreviations: INR*,* international normalized ratio; aPTT*,* activated partial thromboplastin time*,* Hb*,* hemoglobin.*

We analyzed 35 patients contributing 93 ECMO circuit exchanges (median 2, IQR 1–3, range 1–10) over 1317 observation-days. Minor bleeding occurred on 360/1317 observation-days (27.3%, Fig. S8). Major bleeding occurred on 100/1317 observation-days (7.6%, Fig. S5), affecting 29/93 episodes (31.2%) and 21/35 patients (60%). The most common bleeding sites were intrathoracic, soft tissue, and gastrointestinal (Table S2). Major bleeding clustered around circuit exchange (Fig. S4): the pooled bleeding rate within ± 2 days of exchange was 13.1% (61/465 patient-days) versus 4.2% (39/930) outside this window (OR 3.45, 95% CI 2.23–5.39, *p* < 0.001), coinciding with the nadir of coagulation parameters (Fig. 1). Following exchange, bleeding rates declined to 1.1% by days + 3 to + 4, paralleling recovery of coagulation parameters.

The most common indication for circuit exchange was suspected consumptive coagulopathy (42%), followed by gas exchange deterioration and thrombosis-related mechanic dysfunction (Table S3). Systemic thrombotic complications occurred in 22/35 patients (62.9%), including pulmonary embolism (17/35, 48.6%), venous thrombosis (8/35, 22.9%), and other organ infarctions involving the central nervous system, spleen, kidney, or mesentery (7/35, 20.0%). An additional 18 patients who did not undergo circuit exchange were included as a reference group. Among these, 2/18 patients (11%) experienced major bleeding, corresponding to 10/142 observation-days (7.0%); however, substantial differences in ECMO duration and disease severity preclude direct comparison (Figs. S9 and S10). Bleeding risk increased with circuit age (RR 1.08 per day, 95% CI 1.00–1.16; *p* = 0.045) (Table 1).

**Table 1:** Patient characteristics and outcomes by ECMO circuit change status.

Parameter	At least one ECMO circuit change (*n* = 35)	No ECMO circuit change (*n* = 18)
Age (years)	54 (43.5–62)	57.5 (51.5–65)
Male sex, n (%)	9 (25.7%)	13 (72.2%)
Height (cm)	176 (170–180)	175 (170–182)
Weight (kg)	90 (80–100)	100 (91.2–110)
Duration ECMO (hours)	545 (338–917)	214 (142–300)
Duration ECMO (days)	22 (13.5–37.5)	8.5 (5.5–11.8)
ECMO Changes, n	2 (1–3)	0
Major Bleeding, n (%)	21 (60%)	2 (11%)
Intervention required, n (%)	15 (71%)	2 (100%)
Argatroban, n (%)	15 (43%)	3 (17%)
D-dimer (max), mg/L	35.2 (34.4–35.2)	27.4 (8.9–35.2)
Platelet count (min), × 10⁹/L	56 (43–82.5)	92 (47.8–191)
Fibrinogen (min), g/L	2.01 (1.29–2.45)	5.64 (3.43–7.34)
ICU Mortality, n (%)	16 (46%)	10 (56%)
ECMO Mortality, n (%)	13 (37%)	9 (50%)
Cause of death on ECMO, n (%)		
Septic multiorgan failure	9 (69%)	8 (89%)
Hemorrhagic shock	4 (31%)	1 (11%)

Values are median (IQR) or n (%). Comparisons by Wilcoxon rank-sum test (continuous) and Fisher’s exact test (categorical). Causes of hemorrhagic shock included hemothorax (n = 2), retroperitoneal bleeding after femoral artery cannulation (n = 1), and abdominal hemorrhage (n = 1). Interventions included surgical (n = 14) and endoscopic interventions (n = 3). Abbreviations: ECMO, extracorporeal membrane oxygenation; ICU, intensive care unit.

### Biomarker dynamics around ECMO exchange

Biomarker trajectories relative to ECMO circuit exchange are shown in Figs. 1 (coagulation, hemolysis, oxygenator function), S6 (inflammation) and S7 (anticoagulation). Linear mixed model results are summarized in Tables S4A–G and S5.

#### Fibrinogen

Baseline fibrinogen was 4.57 g/L. During the pre-exchange phase, fibrinogen declined by 0.51 g/L/day (β = −0.51, 95% CI − 0.65 to − 0.37, *p* < 0.001), with accelerating decline (β_2_ = − 0.055, *p* < 0.001). While no immediate post-exchange shift occurred (*p* = 0.071), the time-by-phase interaction was significant (β = +0.97, 95% CI 0.68 to 1.25, *p* < 0.001), yielding a post-exchange slope of + 0.46 g/L/day indicating trajectory reversal and fibrinogen recovery. Major bleeding and higher exchange number were associated with lower fibrinogen (Table S4A).

#### D-dimer

D-dimer increased steeply pre-exchange (+ 3.97 mg/L/day, 95% CI 3.02 to 4.91, *p* < 0.001) with accelerating rise. Circuit exchange produced an immediate reduction (− 11.6 mg/L, 95% CI − 13.8 to − 9.4, *p* < 0.001) and profound trajectory reversal (time-by-phase interaction − 5.75, 95% CI − 7.72 to − 3.78, *p* < 0.001; post-exchange slope − 1.78 mg/L/day). Major bleeding was associated with higher D-dimer (+ 3.92, *p* = 0.001), while exchange number had no effect (Table S4B).

#### Platelet count

Platelets declined pre-exchange (− 7.15 × 10⁹/L per day, 95% CI − 11.6 to − 2.7, *p* = 0.002) without significant acceleration. While no immediate post-exchange shift occurred (*p* = 0.206), the time-by-phase interaction was significant (β = +11.57, 95% CI 2.3 to 20.8, *p* = 0.014), yielding a post-exchange slope of + 4.42 × 10⁹/L per day. Major bleeding was associated with substantially lower platelet counts (β = −19.8 × 10⁹/L, *p* < 0.001).

INR showed pre-exchange deterioration with post-exchange recovery; aPTT was stable pre-exchange but showed an immediate reduction following exchange; free hemoglobin, a marker of circuit-induced hemolysis, showed trajectory reversal following exchange (Fig. 1, Table S4G). Hemoglobin declined pre-exchange and recovered post-exchange, with major bleeding associated with substantially lower levels (β = −0.81, *p* < 0.001; Table S4F).

### Predictors of major bleeding

**Fig. 2:**
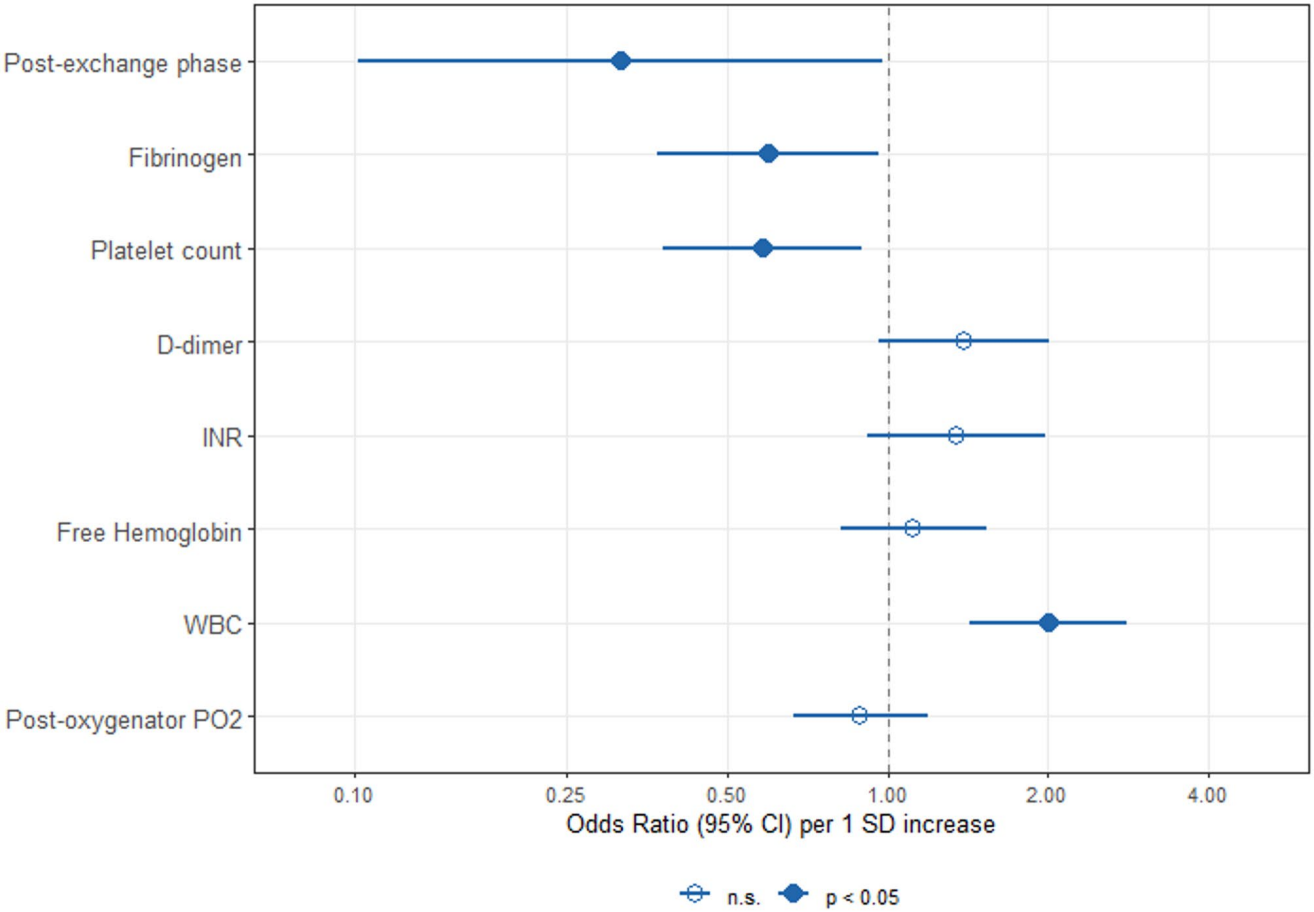
Independent risk factors for major bleeding during ECMO support.

In multivariable analysis adjusting for time and intervention phase, white blood cell count (WBC) was the strongest independent risk factor for major bleeding (OR 2.00 per 1 SD increase, 95% CI 1.43–2.82, *p* < 0.001). Fibrinogen (OR 0.60, 95% CI 0.37–0.96, *p* = 0.035) and platelet count (OR 0.58, 95% CI 0.38–0.90, *p* = 0.014) were independently protective (Fig. 2). The post-exchange phase was associated with a 68% reduction in bleeding risk (OR 0.32, 95% CI 0.10–0.98, *p* = 0.046). D-dimer showed a non-significant trend toward increased risk (OR 1.39, 95% CI 0.96–2.00, *p* = 0.079), while INR, free hemoglobin, and post-oxygenator PO₂ were not independently associated with bleeding (Table S6). In phase-stratified analyses (Table S9), WBC remained significantly associated with bleeding in both phases, whereas fibrinogen and platelet count were predictive only during the post-exchange period (fibrinogen: OR 0.41, 95% CI 0.17–0.99, *p* = 0.047; platelet count: OR 0.30, 95% CI 0.12–0.74, *p* = 0.009). FMI was minimal (< 0.10) for most predictor variables and low to moderate (0.16–0.17) for INR, free hemoglobin, and post-oxygenator PO₂ (Table S7).

Forest plot showing odds ratios (95% confidence intervals) from multivariable generalized linear mixed models with nested random intercepts for episodes within patients. Laboratory predictors are z-standardized; odds ratios represent the change in bleeding risk per 1 standard deviation increase. Filled circles indicate *p* < 0.05; open circles indicate non-significant associations. The dashed vertical line represents OR = 1 (no effect). *Abbreviations: CI*,* confidence interval; INR*,* International Normalized Ratio; PO*_*2*_, *partial pressure of oxygen; SD*,* standard deviation; WBC*,* white blood cell count.*

### Temporal dynamics of major bleeding

**Fig. 3:**
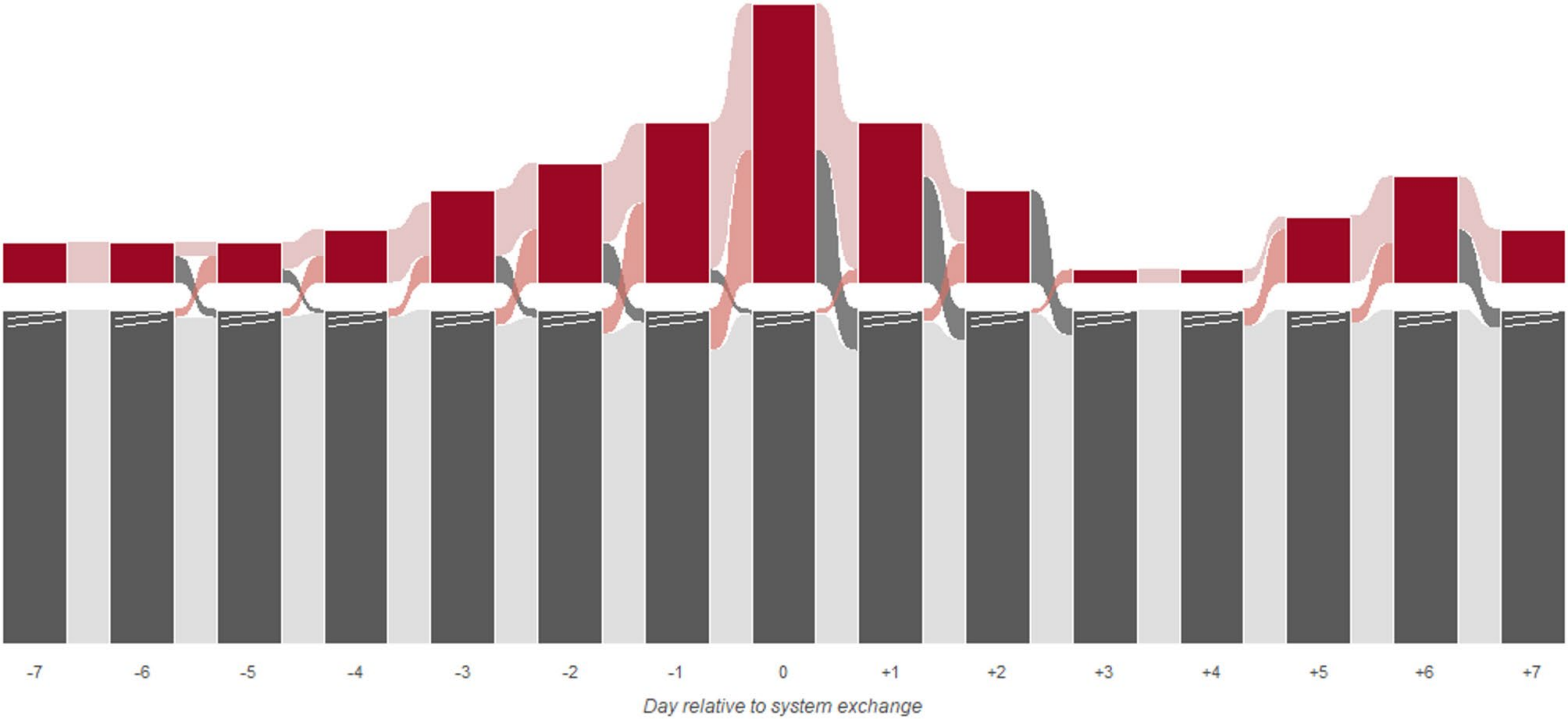
Daily bleeding state transitions across the 15-day observation period centered on ECMO circuit exchange. Dark gray bars represent the number of cases without major bleeding (truncated for display; break marks indicate axis truncation); dark red bars represent cases with major bleeding on each day. Ribbons connecting consecutive days indicate transitions between states, with width proportional to the number of cases. Light gray: persistent non-bleeding (0→0); light rose: persistent bleeding (1→1); red: incident bleeding (0→1); dark gray ribbons: bleeding resolution (1→0).

To capture day‑to‑day bleeding dynamics, we modeled daily transitions between non‑bleeding and bleeding as a two‑state Markov process across 1,224 day‑to‑day transitions from 93 exchange cases in 35 patients (Fig. 3) as a post-hoc analysis. We identified 42 discrete major bleeding episodes in 29 cases (31.2%), with a median duration of 2 days (IQR 1–3). The non‑bleeding state was highly stable (daily persistence 96.6%), whereas the bleeding state showed moderate persistence (60.4%). In the continuous-time multi-state Markov model, ECMO circuit exchange was associated with shorter bleeding episodes: estimated mean duration decreased from 2.7 days (95% CI 1.7–4.2) pre-exchange to 1.2 days (95% CI 0.7–1.9) post-exchange (hazard ratio for bleeding resolution 2.23, 95% CI 1.16–4.29, *p* = 0.017). After adjustment for D-dimer, fibrinogen, and free hemoglobin, the association with faster bleeding resolution remained significant (HR 2.31, 95% CI 1.12–4.74, *p* = 0.023), whereas effects on new bleeding onset were directionally similar but not statistically significant (HR 0.69, 95% CI 0.33–1.43, *p* = 0.315).

### Predictive value of biomarkers for major bleeding complications

**Fig. 4:**
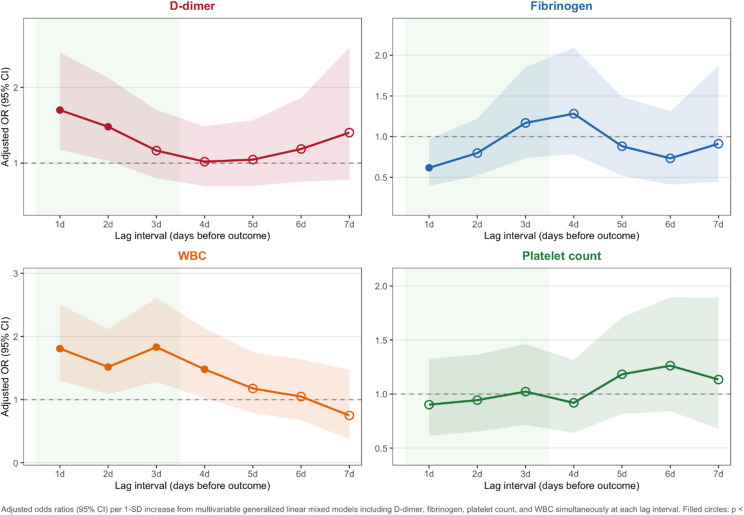
Multivariable time-lagged predictors of major bleeding during ECMO support. Adjusted odds ratios (95% CI) per 1-SD increase from multivariable generalized linear mixed models including D-dimer, fibrinogen, platelet count, and white blood cell count simultaneously at each lag interval. Filled circles indicate *p* < 0.05; open circles indicate non-significant associations. Green shading highlights the 1–3 day predictive window. D-dimer showed a narrow 1–2 day predictive window, whereas WBC demonstrated a broader 1–4 day window, suggesting temporally complementary contributions of coagulopathy and inflammation to bleeding risk. Platelet count was not independently predictive at any lag. All models adjusted for intervention phase and time with nested random intercepts for episodes within patients. Abbreviations: CI, confidence interval; OR, Odds ratio; WBC, white blood cell count.

In exploratory multivariable time-lagged analyses including D-dimer, fibrinogen, platelet count, and white blood cell count simultaneously (Fig. 4, Table S10), D-dimer showed independent predictive value at 1-day (OR 1.70, 95% CI 1.17–2.46, *p* = 0.005) and 2-day lead times (OR 1.48, 95% CI 1.03–2.13, *p* = 0.034), and fibrinogen at lag-1 only (OR 0.62, 95% CI 0.39–0.97, *p* = 0.035). WBC showed a broader predictive window extending to 4 days (lag-1: OR 1.81, 95% CI 1.30–2.50, *p* < 0.001; lag-4: OR 1.48, 95% CI 1.03–2.13, *p* = 0.035). Platelet count was not independently predictive at any lag interval.

### Sensitivity analyses

#### Risk factor models

Comparison of multiple imputation with complete-case analysis showed high concordance in effect direction and significance for the multivariable GLMM of major bleeding risk factors, supporting the missing-at-random assumption (Table S1, Fig. S3).

#### State-transition analysis

Sensitivity GLMMs with nested random intercepts confirmed the Markov findings: the post-exchange phase was associated with reduced odds of incident bleeding (OR 0.14, 95% CI 0.029–0.67, *p* = 0.014) and showed a trend toward reduced bleeding persistence (OR 0.18, 95% CI 0.028–1.14, *p* = 0.069). The attenuation of the persistence effect likely reflects limited statistical power, as only 96 transitions originated from the bleeding state. No individual laboratory parameter was independently associated with either transition type after adjustment for intervention phase.

### Additional exploratory analyses

Exploratory analyses of patients without exchange (*n* = 18) and terminal-phase trajectories (*n* = 22) are presented in Figures S9–S10 and S11–S12.

## Discussion

In this retrospective cohort of patients with COVID-19 ARDS supported with VV-ECMO, trajectories of D-dimer, fibrinogen, and platelets around circuit exchange were consistent with a consumptive coagulopathy that was at least partially reversed after circuit replacement, with major bleeding clustering in the immediate peri-exchange period. White blood cell count emerged as the strongest independent predictor of bleeding in both phases in multivariable models, and time-lagged analyses reinforced this association, with WBC showing a broader predictive window (1–4 days) than individual coagulation parameters. The temporal profiles of the two marker groups were distinct: inflammation-associated risk extended over several days, whereas coagulation derangement signaled imminent hemorrhage within 1–2 days. This suggests that these markers may capture at least partially independent aspects of the pathophysiology underlying bleeding during ECMO support. The post-exchange decline in bleeding prevalence and improvement in coagulation markers support the concept that timely circuit replacement may help interrupt an evolving consumptive process, and exploratory state-transition analyses further suggested faster resolution of ongoing bleeding episodes after exchange.

Our observations align with prior studies reporting improvement of deranged hematological markers and D‑dimer reduction after ECMO circuit exchange^6–8,10,18,19^, supporting consumptive coagulopathy as a key mechanism during extended support^20,21^. Hoffman et al. further highlighted the role of hemolysis markers in this context, reporting a sharp rise in plasma-free hemoglobin before circuit exchange that normalized thereafter^19^. However, granular longitudinal data linking daily biomarker dynamics to bleeding events around circuit exchange remain scarce. Because bleeding risk during ECMO is dynamic rather than static, biomarker trajectories surrounding bleeding events may be more informative than baseline values at ECMO initiation. Our analysis addresses this gap by linking daily coagulation, hemolysis, and inflammatory marker trajectories around circuit exchange to systematically recorded major bleeding events across 93 exchanges in 35 patients, and by characterizing day-to-day bleeding dynamics using state-transition modeling. The 18 patients without circuit exchange in our cohort had substantially lower bleeding rates (11% vs. 60%) and less pronounced coagulation derangements. This likely reflects differences in disease severity and ECMO duration. We therefore refrained from formal comparative analyses and do not interpret this contrast as causal evidence for circuit‑related coagulopathy.

The temporal rise in D-dimer and concurrent decline in fibrinogen and platelet counts preceding major bleeding suggest that these elevations do not simply reflect a stable background coagulopathy, but rather indicate progressive biological activation culminating in consumptive coagulopathy and hemorrhage. In COVID-19 ARDS, this process is further amplified by virus-induced endothelial injury and immunothrombosis, creating a prothrombotic milieu^22,23^ that is intensified by extracorporeal circulation^24–26^. The elevation of free hemoglobin before major bleeding events is consistent with progressive circuit degradation with increasing mechanical hemolysis due to intraluminal thrombus formation. In support of this interpretation, bleeding rates declined rapidly following exchange, paralleled by immediate normalization of D-dimer and gradual recovery of fibrinogen and platelet counts, suggesting that circuit replacement may interrupt the consumptive process.

These observations underscore that coagulopathy in ECMO patients is multifactorial, driven by the interplay of circuit-mediated consumptive coagulopathy, sepsis-associated hemostatic dysregulation, procedural bleeding complications, and progressive circuit degradation. This heterogeneity is illustrated by distinct terminal trajectories: patients dying from hemorrhagic shock exhibited persistently lower fibrinogen and more pronounced thrombocytopenia, whereas patients with septic multiorgan failure maintained higher fibrinogen despite progressive decline, suggesting different dominant pathophysiological pathways.

Bleeding complications are clearly linked to mortality, whereas thrombotic events are not^3^. Yet thrombotic complications remain poorly characterized in observational studies as they require systematic clinical and radiological screening^3^, and the thrombotic state of the ECMO circuit is still essentially a black box. If ECMO-related bleeding is driven in part by thrombotic changes within the circuit, this carries important clinical implications. In consumptive coagulopathy, substitution of coagulation factors and platelets may perpetuate the vicious cycle of consumption if the underlying trigger persists^21,27^. Only elimination of the underlying trigger can resolve the consumptive coagulopathy. Identifying predictors that inform the timing of elective circuit exchange before complications occur is therefore essential, both for clinical decision-making and for the design of future anticoagulation trials.

Time-lagged analyses identified D-dimer and fibrinogen as early warning markers predicting major bleeding 1–2 days in advance, suggesting a narrow but potentially actionable time window for clinical intervention to counteract or prevent major bleeding. This is consistent with Helms et al.^5^, who identified a rising D-dimer over 48 h as a predictor of subsequent bleeding complications. aPTT showed similar predictive timing (Table S10), though its interpretation is confounded by therapeutic heparin anticoagulation. Notably, D-dimer was independently predictive only in the time-lagged model, not in the primary multivariable model of same-day predictors for major bleeding. This may reflect event-driven collinearity with platelet count and fibrinogen, which show pronounced declines during acute major bleeding (with fibrinogen exhibiting accelerating decline) and may therefore absorb shared variance from D-dimer in same-day models. In the lagged analysis, the divergent pre-bleeding trajectories of these coagulation markers are disentangled, which may explain why D-dimer emerged as an independent predictor at 1–2 day lead times while platelet count, which was significant in the same-day model, showed no predictive value at any lag interval. This interpretation requires confirmation in prospective studies with serial high-frequency sampling.

Our data suggest that integrated monitoring of coagulation and inflammatory marker trajectories could inform the development of bleeding risk stratification strategies, which are currently lacking for ECMO patients. Given that major hemorrhage rates remain substantial even in contemporary cohorts^2,3^, individualized hemostatic management rather than a uniform approach to anticoagulation appears essential.

A key strength of this study is the dense longitudinal data structure, with daily laboratory measurements systematically linked to daily bleeding status across 93 exchange episodes, enabling the characterization of dynamic biomarker-bleeding relationships that are difficult to capture in cross-sectional or aggregate study designs. The multivariable time-lagged analysis extends prior work by identifying independent, temporally distinct predictive contributions of coagulation and inflammatory markers, and by demonstrating that the predictive relevance of individual markers differs depending on whether same-day or time-lagged associations are assessed. Internal validity is further supported by the homogeneous cohort of COVID-19 ARDS patients treated at a single high-volume center with protocolized laboratory monitoring and standardized anticoagulation management, and by the use of established ELSO criteria for major bleeding classification. Statistical methods accounting for the hierarchical data structure, repeated measurements, and missing data support the validity of the estimates.

Several limitations warrant consideration. First, the single-center, retrospective design with a modest sample size (35 patients, 93 exchanges, 100 bleeding observation-days) limits statistical power for subgroup analyses, and all multivariable and state-transition models should be interpreted as hypothesis-generating. Second, ECMO circuit exchange was triggered by clinical judgment in the context of suspected circuit degradation rather than by a strict protocol, introducing confounding by indication. Our analyses therefore cannot establish a causal benefit of exchange but only support the hypothesis that timely replacement may modify an ongoing consumptive process. Third, the multivariable GLMM included 10 fixed effects, corresponding to an events-per-variable ratio of approximately 10. While this meets the conventional rule-of-thumb for standard logistic regression, its direct applicability to GLMMs with repeated measures is uncertain, because these models exploit the full longitudinal data structure (1317 observation-days) rather than relying solely on the number of events. To limit the risk of overfitting and preserve model stability, we restricted predictors to variables with strong pathophysiological justification and excluded anticoagulation monitoring levels, which showed no meaningful between-group differences (Fig. S7). The multivariable time-lagged models were further restricted to four predictors given the reduced sample size inherent to lagged analyses. aPTT was excluded from all multivariable models because of confounding by therapeutic anticoagulation. The multi-state Markov models did not account for within-patient clustering, assumed time-homogeneous transition intensities, and were restricted to complete cases. Both analytical approaches require confirmation in larger cohorts. Fourth, the homogeneous COVID-19 ARDS population with pronounced inflammatory and thrombotic activation likely amplifies consumptive coagulopathy patterns. Whether similar biomarker trajectories and bleeding associations apply to non-COVID ARDS or other ECMO indications remains to be determined.

## Conclusions

Taken together, our results suggest that bleeding risk during prolonged VV-ECMO in COVID-19 ARDS is associated with two temporally distinct biomarker patterns: rising D-dimer and declining fibrinogen 1–2 days before hemorrhage, consistent with consumptive coagulopathy, and white blood cell count elevations extending up to 4 days before onset, consistent with systemic inflammation. These temporally complementary biomarker patterns may define a potential monitoring window preceding major hemorrhage. Circuit exchange was associated with reversal of coagulation trajectories and shorter bleeding duration, suggesting that timely replacement may interrupt an evolving consumptive process. Future multicenter, prospective studies in broader ARDS populations are needed to validate these findings and to evaluate whether integrated monitoring of coagulation dynamics and inflammatory status can improve risk stratification and guide individualized anticoagulation and circuit management strategies.

## Supplementary Information

Below is the link to the electronic supplementary material.

Supplementary Material 1

## Data Availability

All data generated or analyzed during this study are included in this published article and its supplementary information files. The individual-level datasets are available from the corresponding author on reasonable request.

## Abbreviations

anti-Xa: Anti-factor Xa activity
ARDS: Acute respiratory distress syndrome
aPTT: Activated partial thromboplastin time
CI: Confidence interval
COVID-19: Coronavirus disease 2019
DRKS: Deutsches Register Klinischer Studien (German Clinical Trials Register)
ECMO: Extracorporeal membrane oxygenation
ELSO: Extracorporeal Life Support Organization
FDR: False discovery rate
FMI: Fraction of missing information
GLMM: Generalized linear mixed model
Hb: Hemoglobin
HR: Hazard ratio
ICU: Intensive care unit
INR: International normalized ratio
IQR: Interquartile range
LMM: Linear mixed model
MCAR: Missing completely at random
MICE: Multiple imputation by chained equations
OR: Odds ratio
PMM: Predictive mean matching
PO₂: Partial pressure of oxygen
SD: Standard deviation
STROBE: Strengthening the Reporting of Observational Studies in Epidemiology
VIF: Variance inflation factor
VV-ECMO: Venovenous extracorporeal membrane oxygenation
WBC: White blood cell count
