# Supplementary material for: Daily biomarker trajectories predict major bleeding in patients on venovenous ECMO for ARDS: a retrospective longitudinal cohort study
Source: Sci Rep. 2026 Apr 9;16:12041. doi: 10.1038/s41598-026-47560-0 (PMC13069085; doi:10.1038/s41598-026-47560-0)
Supplement: Supplementary file 1 — Supplementary Material 1 [file 41598_2026_47560_MOESM1_ESM.docx]

**Online Data Supplement**

**Daily Biomarker Trajectories Predict Major Bleeding in Patients on Venovenous ECMO for ARDS: A Retrospective Longitudinal Cohort Study**

Thomas Stueber*, Jil-Madeline Homeier*, Hans-Joerg Gillmann, Jona Wassong, Carolin Jung
*contributed equally

**Table of Contents page**

**A – Data Structure and Preprocessing**

1. **Detailed description of Data Structure and Preprocessing 3-8**
2. **Figure S1. Pairwise Pearson correlation matrix of laboratory 9
   parameters**
3. **Table S1. Comparison of observed and imputed values. 10**
4. **Figure S2. Density plots comparing observed and imputed 11
   distributions.**
5. **Figure S3. Sensitivity analysis comparing multiple imputation 12
   versus complete case analysis**

**B – Supplementary material of the Main analysis**

1. **Figure S4. Daily major bleeding rate in patients undergoing ECMO 13**

**circuit exchange.**

1. **Table S2. Distribution of major bleeding sites across affected episodes 14**

**and patients.**

1. **Table S3. Documented indications for ECMO circuit exchange. 15**
2. **Figure S5. Heatmap of major bleeding events by case and day in 16-17**

**patients undergoing ECMO circuit exchange.**

1. **Figure S6. Inflammatory marker trajectories around ECMO circuit 18**

**exchange.**

1. **Figure S7. Anticoagulation monitoring parameters around ECMO 19-20**

**circuit exchange, stratified by major bleeding status.**

1. **Figure S8. Daily minor bleeding rates across the 15-day observation 20
   period centered on ECMO circuit exchange.**
2. **Tables S4A-G. Linear mixed model results for longitudinal trajectories 21-24
   of laboratory parameters surrounding ECMO circuit exchange.**
3. **Table S5. Random effects variance components from linear mixed models. 24**
4. **Table S6. Independent risk factors for major bleeding during ECMO support. 25**
5. **Table S7. Fraction of missing information (FMI) for multivariable model 25 parameters.**
6. **Table S8. Time-lagged predictors of major bleeding during ECMO 26-27
   support.**
7. **Table S9. Phase-stratified predictors of major bleeding during ECMO 27
   support.**
8. **Table S10. Time-lagged predictors of major bleeding (multivariable 28
   GLMMs)**

**C – Additional analyses**

1. **Figure S9. Laboratory parameter trajectories in patients with D-dimer 29
   > 35 mg/L without ECMO circuit exchange.**
2. **Figure S10. Temporal distribution of major bleeding events and blood 30**

**product transfusions in patients with D-dimer > 35 mg/L without ECMO**

**circuit exchange.**

1. **Figure S11. Laboratory parameter trajectories in the 7 days preceding 31-32
   death, stratified by cause of death.**
2. **Figure S12. Temporal distribution of major bleeding events and blood 33**

**product transfusions in deceased ECMO patients.**

1. **Detailed description of Data Structure and Preprocessing**

Patient-Case Hierarchy

The study comprised a three-level hierarchical structure: 35 unique patients contributed 93 ECMO circuit exchange cases, yielding 1,317 daily observations after preprocessing. Each ECMO circuit exchange was treated as a separate analytical case to maintain temporal independence. Patient distribution across exchanges was as follows: 15 patients (42.9%) with 1 exchange, 8 patients (22.9%) with 2 exchanges, 6 patients (17.1%) with 3 exchanges, and 6 patients (17.1%) with 5–10 exchanges. One patient contributed 10 exchanges (maximum). This hierarchical structure required statistical methods accounting for both within-patient correlation across multiple exchanges and within-case correlation across daily measurements.

Temporal Structure

Each case encompassed a 15-day observation window centered on the ECMO circuit exchange event (day 0). Laboratory measurements were obtained daily from Day -7 through Day +7, with the physical exchange occurring on Day 0 immediately following that day's measurements. We defined the pre-intervention phase as Days -7 to 0 (n=648 observations, 59.2%) and the post-intervention phase as Days +1 to +7 (n=447 observations, 40.8%). This design enabled within-case comparison of biomarker trajectories before and after circuit exchange.

### Missing Data Handling

### Structural Exclusions: Of 1,395 total observations (93 ECMO circuit exchange episodes × 15 days), 78 (5.6%) were considered structurally missing and excluded prior to imputation: 51 observation time points (3.7%) fell outside the observation window because they occurred after death or ECMO explantation, and 27 observation time points (1.9%) fell before ICU admission. Several laboratory parameters (free hemoglobin, post-oxygenator PO₂, D-dimer, Fibrinogen) were measured exclusively during extracorporeal support and therefore unavailable after explantation. These observations represent data that could not exist rather than data missing at random.

### Missing Data Patterns: Among the 1,317 eligible observations, missing data rates for laboratory parameters were as follows:

| Parameter | Missing observations |
| --- | --- |
| Post-oxygenator PO₂ | 304 (23.1%) |
| Free hemoglobin | 92 (7%) |
| Fibrinogen | 61 (4.6%) |
| Ferritin | 41 (3.1%) |
| D-dimer | 23 (1.7%) |
| INR | 18 (1.4%) |
| CRP | 18 (1.4%) |
| White blood cell count | 15 (1.1%) |
| Platelet count | 15 (1.1%) |
| aPTT | 13 (1%) |

### Little's MCAR test was statistically significant (χ² = 214.44, df = 104, p < 0.0001), indicating that the MCAR assumption was violated and that missingness was related to observed variables. Based on this finding, we employed multiple imputation under the assumption of data missing at random (MAR). Although MAR cannot be empirically verified, this assumption is plausible given that missingness was primarily driven by clinical factors captured in the dataset (e.g., timing relative to ICU admission, laboratory ordering patterns). The validity of this assumption was further supported by concordance between multiple imputation and complete case analysis results in sensitivity analyses.

### Multiple Imputation

Missing laboratory values were imputed using predictive mean matching (PMM), which preserves observed data distributions and generates biologically plausible imputed values by drawing from observed donor values with similar predicted means. The imputation model included 11 laboratory parameters: D-dimer, fibrinogen, platelet count, hemoglobin, free hemoglobin (fHb), post-oxygenator PO₂, INR, aPTT, ferritin, C-reactive protein (CRP), and white blood cell count (WBC). Auxiliary variables included time (Days), intervention phase, exchange number, and the outcome variable (major bleeding). All laboratory parameters were allowed to predict each other, leveraging biological correlations to improve imputation accuracy. Of these, 7 parameters (D-dimer, fibrinogen, platelet count, INR, fHb, WBC, post-oxygenator PO₂) were subsequently used as predictors in the multivariable GLMM, selected a priori based on pathophysiological relevance. Procalcitonin was excluded from imputation due to excessive missingness (>50%).

We generated 30 multiply imputed datasets with 20 iterations each, using a fixed random seed for reproducibility. Convergence was assessed visually through trace plots (Figure S2) and Gelman-Rubin diagnostics (all R̂ < 1.1). Mean differences between observed and imputed values were <1% for all parameters (Table S1). Results from each imputed dataset were combined using Rubin's rules.

Statistical Models_

**Linear Mixed Models for Biomarker Trajectories:** Coagulation, hematologic, and hemolysis parameters were analyzed using linear mixed models with nested random intercepts for episodes within patients. Fixed effects included centered time (days from exchange) as a linear term, a quadratic term to account for non-linear trajectory components, intervention phase as a binary indicator to estimate the immediate level shift following exchange, and an interaction term between centered time and intervention phase to assess whether the slope of biomarker change differed between phases. Major bleeding occurrence and sequential exchange number were included as additional fixed effects. Model fit was assessed using intraclass correlation coefficients (ICC). All models were fitted using the bobyqa optimizer (maximum function evaluations = 100,000) on each of the 30 imputed datasets, with results pooled using Rubin's rules.

**Generalized Linear Mixed Models for Bleeding Risk Prediction**: The association between laboratory parameters and major bleeding was assessed using generalized linear mixed models (GLMM) with a binomial distribution and logit link function. Models included nested random intercepts for episodes within patients to account for repeated measurements and the hierarchical data structure. Time was modeled using natural cubic splines with 2 degrees of freedom centered at the exchange day to allow for non-linear temporal trends. Fixed effects included intervention phase and z-standardized laboratory predictors; odds ratios represent the change in bleeding risk per 1 standard deviation increase. Models were fitted across 30 imputed datasets and pooled using Rubin's rules. To assess whether laboratory predictors differed in their association with bleeding between phases, the multivariable GLMM was additionally fitted separately for the pre-exchange and post-exchange periods, excluding the intervention term.

Exploratory Analyses

**Time-Lagged Analyses.** To assess temporal relationships between biomarkers and subsequent bleeding, we fitted multivariable time-lagged GLMMs at each lag interval (1–7 days), including D-dimer, fibrinogen, platelet count, and white blood cell count simultaneously as z-standardized lagged predictors. These four parameters were selected based on their significant or borderline-significant associations in the concurrent multivariable model (Table S6); INR, free hemoglobin, and post-oxygenator PO₂ were excluded as they showed no independent concurrent association with bleeding. Activated partial thromboplastin time was excluded from all multivariable models because of confounding by therapeutic anticoagulation. Lagged predictors were generated within episodes to preserve temporal ordering and prevent information leakage. All models were adjusted for intervention phase and time (natural cubic splines, df = 2) with nested random intercepts for episodes within patients, fitted across 30 imputed datasets and pooled using Rubin's rules. Univariate time-lagged analyses for all eight parameters are reported in Table S8.

**State-Transition Analysis of Bleeding Dynamics.** In post-hoc analyses motivated by the observed temporal clustering of bleeding around circuit exchange, daily bleeding status was modeled as a two-state Markov process (0 = no major bleeding, 1 = major bleeding). A continuous-time multi-state Markov model (msm package, version 1.7.1) was used to estimate transition intensities and mean sojourn times. Three nested models were compared using likelihood ratio tests: (1) a baseline model, (2) an intervention model with circuit exchange as time-varying covariate, and (3) a full model additionally adjusting for z-standardized D-dimer, fibrinogen, and free hemoglobin. The restriction to three laboratory covariates (compared with seven in the GLMM) was necessary to ensure convergence. The full model used complete cases (92.1% of observations), as msm does not support multiple imputation. Hazard ratios were derived from log-linear covariate effects; p-values were computed using Wald z-tests.

Sensitivity Analyses

**Imputation Robustness***.* To assess the robustness of findings to the handling of missing data, we compared results from the multiple imputation approach with complete case analysis (CCA; n = 954 observations, 72.4% of eligible observations). Generalized linear mixed models with identical specifications were fitted to both datasets. Concordance of effect estimates and statistical significance across both approaches supported the validity of the MAR assumption (Table S1 and Figure S3).

**State-Transition Model Validation.** As the continuous-time multi-state Markov model does not accommodate random effects for hierarchically clustered observations, we additionally fitted GLMMs with binomial distribution and logit link to the transition data. Separate models were fitted for each transition type: (a) incident bleeding, conditional on no bleeding at day t, and (b) persistent bleeding, conditional on bleeding at day t. Models included intervention phase, day, sequential exchange number, and z-standardized laboratory covariates as fixed effects, with nested random intercepts for episodes within patients. GLMMs were fitted across 30 multiply imputed datasets and pooled using Rubin's rules.

Model Diagnostics

The fraction of missing information (FMI) ranged from 0.03 to 0.16 across multivariable GLMM parameters, indicating minimal impact of imputation on effect estimates (Table S6). Multicollinearity diagnostics confirmed no concerning correlations among predictors (all VIF < 1.5, all |r| < 0.40).

**Figure S1. Correlation matrix of laboratory parameters.**


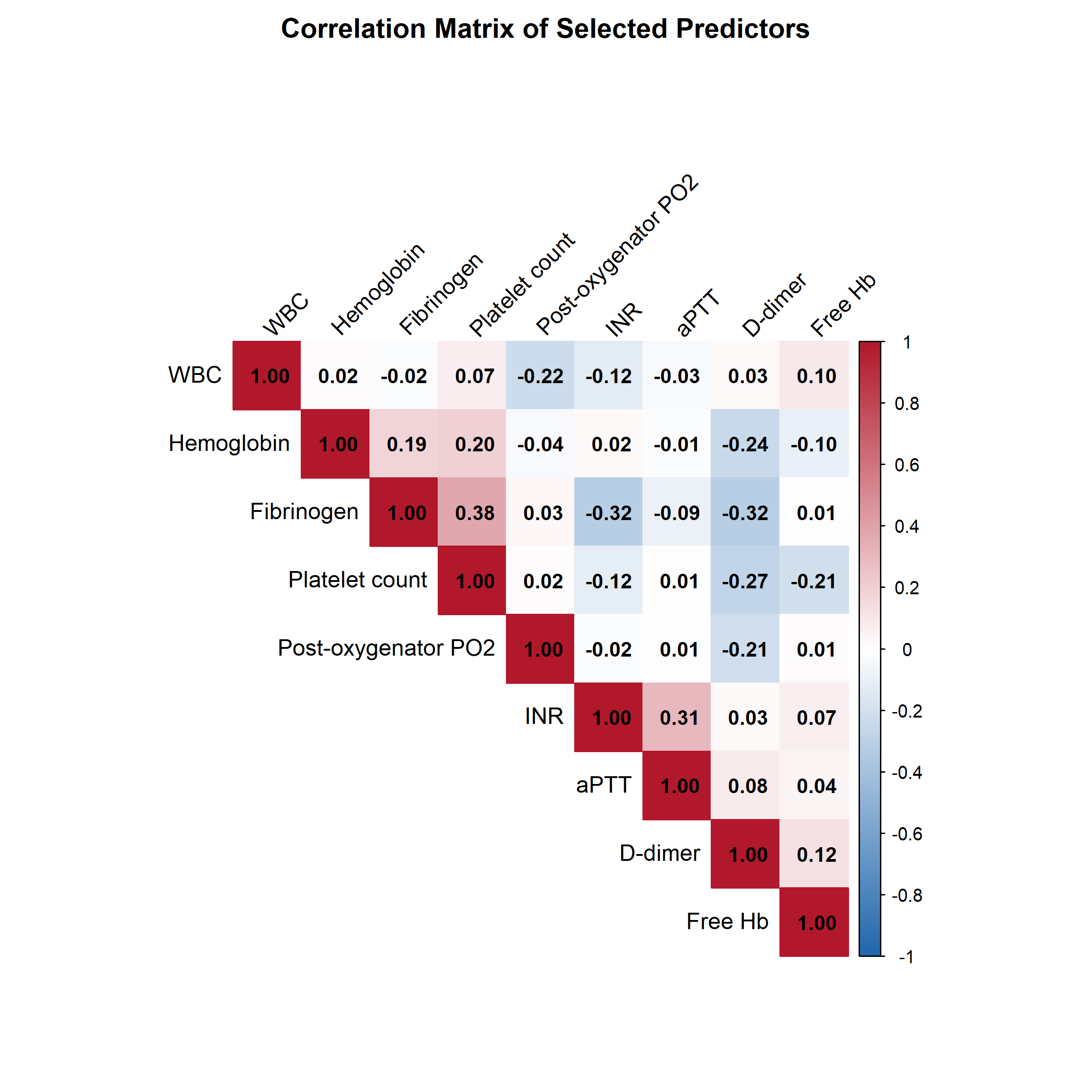


Pairwise Pearson correlation coefficients among coagulation and hematologic parameters (n = 1,317 observations). Variance inflation factors were <5 for all parameters. *Abbreviations: aPTT, activated partial thromboplastin time; Hb, hemoglobin; INR, international normalized ratio; PO_2_, partial pressure of oxygen; WBC, white blood cell count.*

**Table S1. Comparison of observed and imputed values.**

| **Variable** | **Observed Mean** | **Complete Case Mean** | **Imputed Mean** | **Difference  MI vs. Obs (%)** |
| --- | --- | --- | --- | --- |
| D-dimer | 17.55 | 18.29 | 17.52 | -0.2 |
| Fibrinogen | 3.99 | 3.87 | 4.02 | +0.9 |
| Platelet count | 121.75 | 119.24 | 121.61 | -0.1 |
| Hemoglobin | 9.05 | 9.02 | 9.05 | 0.0 |
| Free hemoglobin | 95.03 | 98.47 | 94.81 | -0.2 |
| Oxygenator PO_2_ | 463.61 | 462.76 | 464.70 | +0.2 |
| INR | 1.21 | 1.21 | 1.21 | +0.1 |
| aPTT | 56.22 | 56.40 | 56.20 | 0.0 |
| Ferritin | 1509.23 | 1503.09 | 1510.47 | +0.1 |
| CRP | 110.58 | 108.90 | 110.81 | +0.2 |
| WBC | 13.19 | 13.28 | 13.20 | +0.1 |

*Abbreviations: CRP, C-reactive protein; INR, international normalized ratio; MI, multiple imputation; Obs, observed; aPTT, activated partial thromboplastin time; PO_2_, partial pressure of oxygen; WBC, White blood cell count.*

**Figure S2. Density plots comparing observed and imputed distributions.**

**
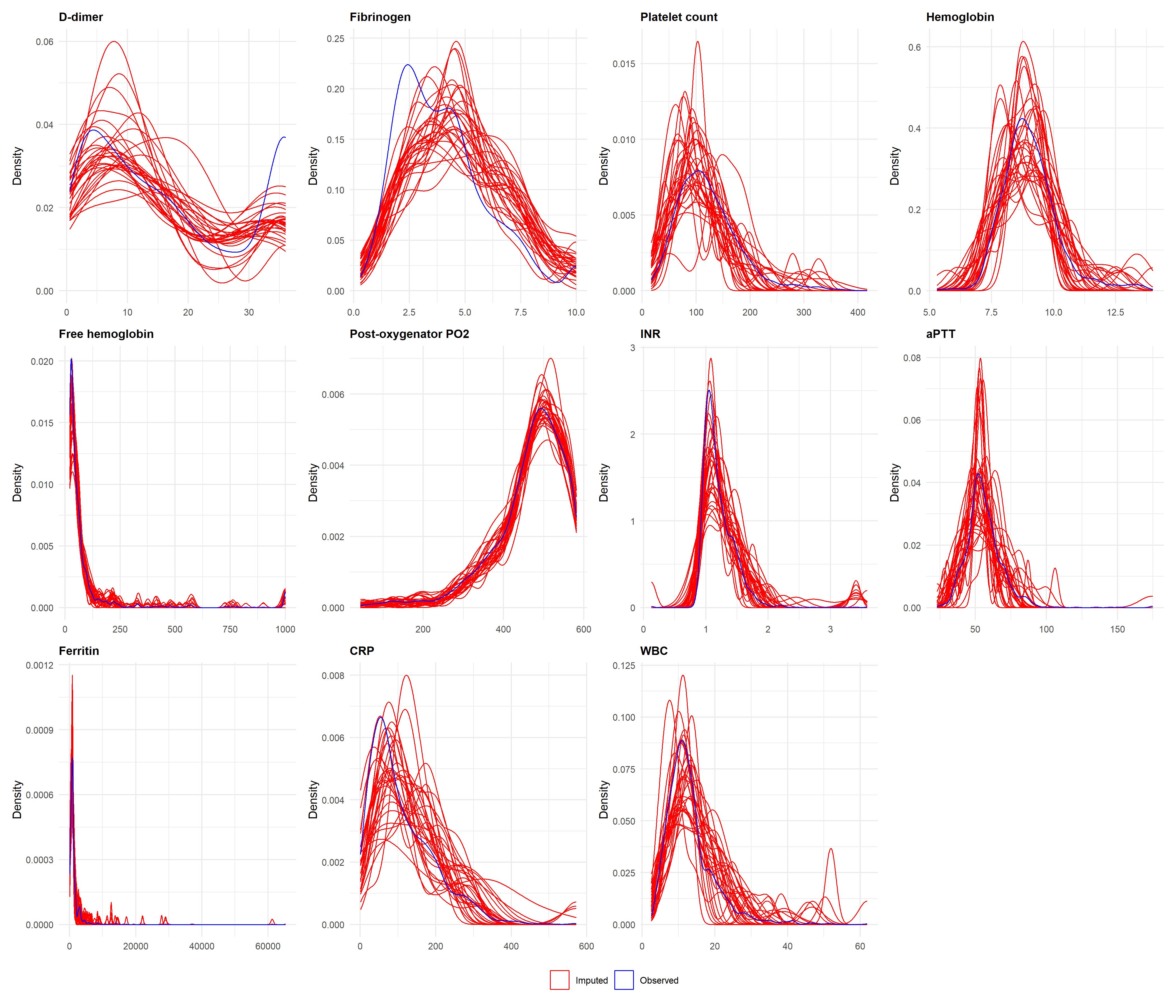
**

Blue lines represent observed data; red lines represent imputed values across 30 imputed datasets. Close overlap indicates that predictive mean matching preserved the original data distributions. All imputed values fall within physiologically plausible ranges. *Abbreviations: CRP, C-reactive protein; Fib, fibrinogen; fHb, free hemoglobin; Hb, hemoglobin; INR, international normalized ratio; PO_2_, oxygen partial pressure; aPTT, activated partial thromboplastin time, WBC, white blood cell count.*

**Figure S3. Sensitivity analysis comparing multiple imputation versus complete case analysis**

**
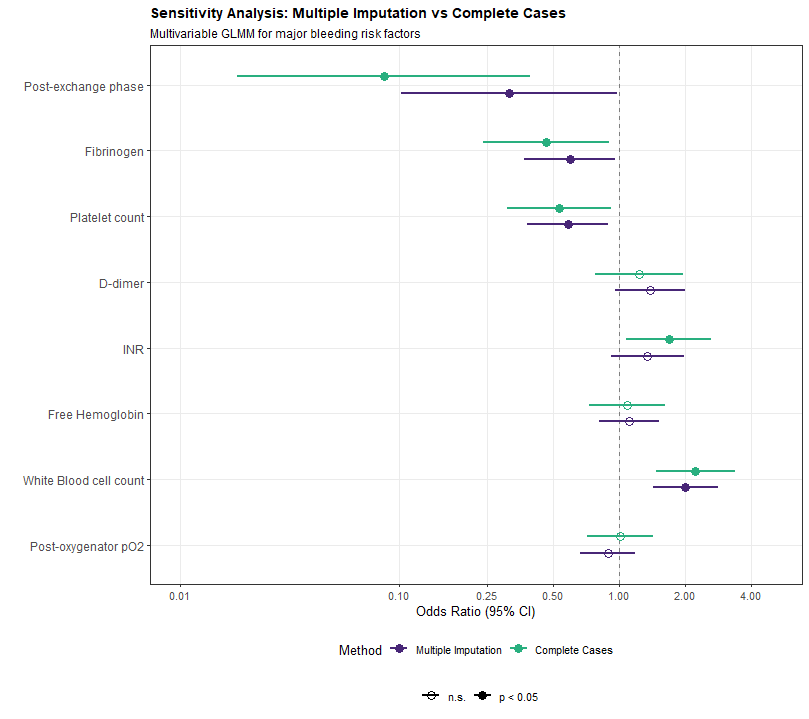
**

Odds ratios (95% CI) for major bleeding risk factors from multivariable GLMMs. Purple: multiple imputation (30 datasets); teal: complete case analysis. Filled symbols, p < 0.05. All predictors z-standardized. High concordance between methods supports robustness of findings and validity of the missing at random assumption. Random effects for patient and measurement nested within patient; temporal adjustment via natural splines (df = 2). *Abbreviations: INR, international normalized ratio; PO_2_, oxygen partial pressure.*

**Figure S4. Daily major bleeding rate in patients undergoing ECMO circuit exchange.**


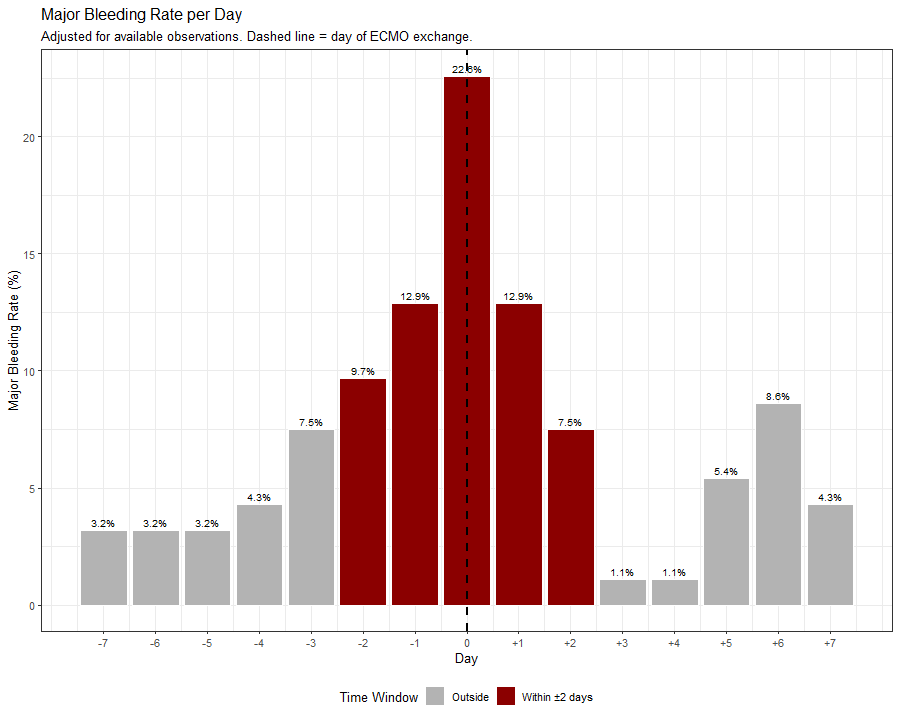


Bars represent the proportion of observed patient-days with major bleeding events for each day of the 15-day observation period. Rates are adjusted for available observations (censoring due to death, ECMO decannulation, or subsequent circuit exchange). Dark red bars indicate the ±2-day window around circuit exchange, gray bars indicate days outside this window. The dashed vertical line marks the day of ECMO circuit exchange (Day 0).

**Table S2. Distribution of major bleeding sites across affected episodes and patients.**

| **Bleeding site** | **Case (n=29)** | **Patient (n=23)** |
| --- | --- | --- |
| Intrathoracic | 10 | 8 |
| Gastrointestinal | 4 | 4 |
| Central nervous system | 1 | 1 |
| Retroperitoneal | 3 | 3 |
| Tissue | 6 | 5 |
| Diffuse | 5 | 2 |

Each episode (n = 29) and patient (n = 23) was classified according to the predominant site of the qualifying major bleeding event. Patient counts include 21 patients from the circuit exchange group and 2 patients from the non-exchange group. Categories are mutually exclusive. Intrathoracic bleeding includes tracheobronchial and thoracal bleeding; tissue bleeding refers to space-occupying soft tissue hemorrhages (spontaneous or post-puncture) requiring surgical evacuation; diffuse bleeding denotes clinically overt hemorrhage without a single predominant source, including bleeding from multiple sites or surgical wounds.

**Table S3. Documented indications for ECMO circuit exchange.**

| **Documented indications for Circuit exchange** | **All exchanges (n = 93)** |
| --- | --- |
| Suspected consumptive coagulopathy | 39 (41.9%) |
| Oxygenator gas exchange deterioration | 20 (21.5%) |
| Thrombosis-related ECMO dysfunction | 17 (18.3%) |
| Hemolysis | 16 (17.2%) |
| Mechanical equipment failure | 1 (1.1%) |

Values are n (%). Indications were extracted from medical records and classified into the primary documented reasoning for each exchange. Thrombosis-related ECMO dysfunction includes reduced blood flow and increased transmembrane pressure gradient. Categories are mutually exclusive. Where multiple indications were documented, the primary clinical indication as recorded by the treating physician was used.

**Figure S5. Individual bleeding trajectories across the 15-day observation period centered on ECMO circuit exchange.**

**
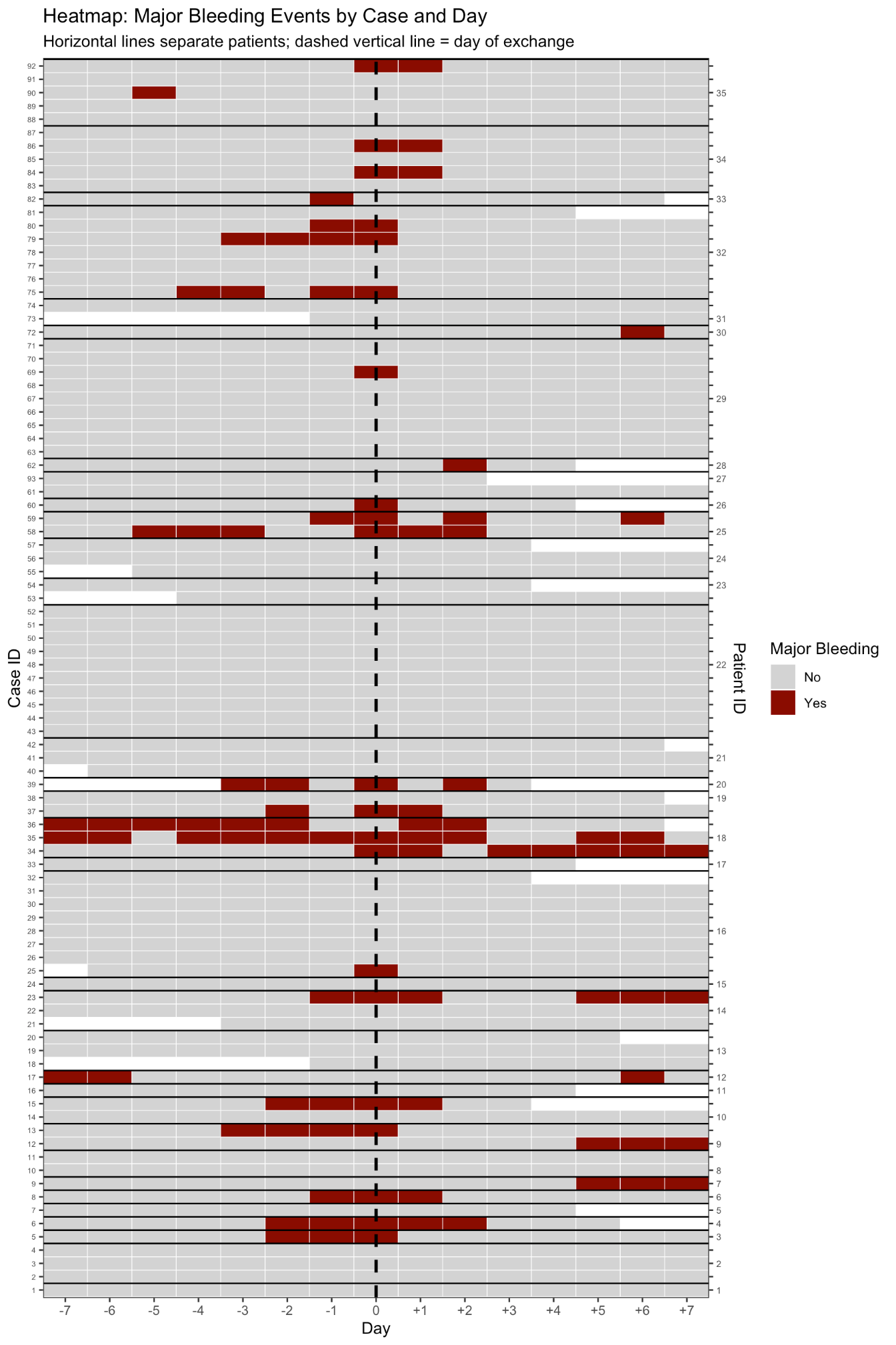
**

**Each row represents one circuit exchange episode (n = 93, left axis), grouped by patient (n = 35, right axis; horizontal lines separate patients). Columns represent days relative to circuit exchange (day 0, dashed vertical line). Dark red tiles indicate days with major bleeding (per ELSO criteria); gray tiles indicate days without major bleeding; white spaces denote structurally missing observations (before ICU admission, after death, or after ECMO explantation). Episodes are ordered by patient, illustrating both within-patient recurrence of bleeding across successive exchanges and the temporal clustering of bleeding events around the day of circuit exchange.**

**Figure S6. Inflammatory marker trajectories around ECMO circuit exchange, stratified by major bleeding status.**


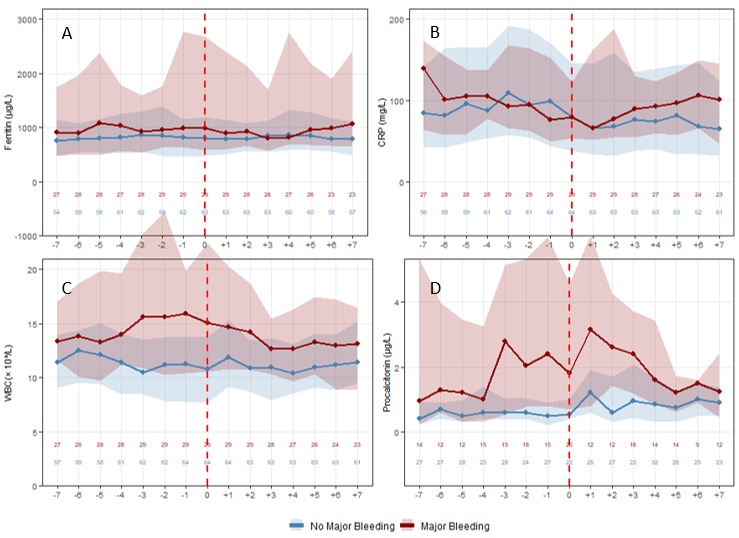


Ferritin (A), C-reactive protein (B), white blood cell count (C), and procalcitonin (D) trajectories from day −7 to day +7 relative to circuit exchange (day 0, dashed line). Data from 93 ECMO circuit exchange episodes in 35 patients, stratified by occurrence of major bleeding (red, n = 29) versus no major bleeding (blue, n = 64). Lines represent medians; shaded areas, interquartile ranges. Numbers denote sample size at each time point. WBC was consistently higher in episodes with major bleeding throughout the observation period. Ferritin and CRP showed wide interquartile ranges with substantial overlap between groups. Procalcitonin showed considerable missingness, limiting interpretability. *Abbreviations: CRP, C-reactive protein; WBC, white blood cell count.*

**Figure S7. Anticoagulation monitoring parameters around ECMO circuit exchange, stratified by major bleeding status.**


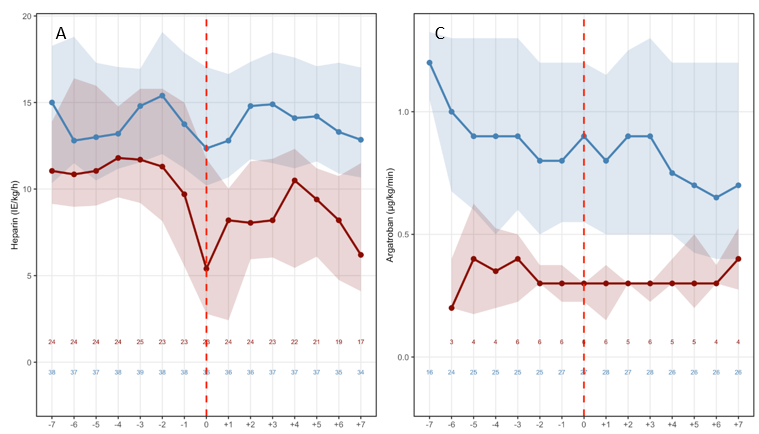

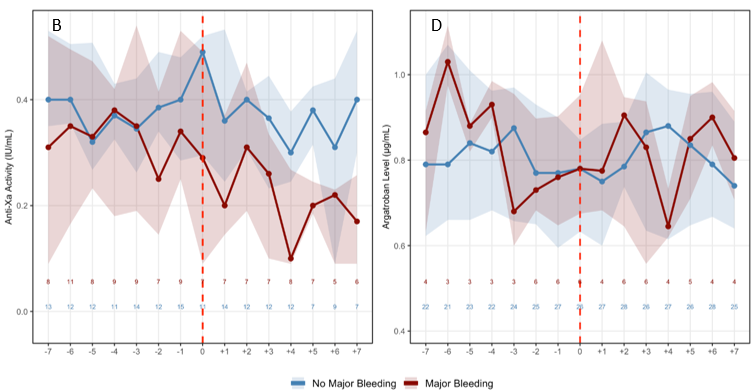


Heparin dose (A), anti-Xa activity (B), argatroban dose (C), and argatroban level (D) from day −7 to day +7 relative to circuit exchange (day 0, dashed line). Data from 93 ECMO circuit exchange episodes in 35 patients, stratified by occurrence of major bleeding (red) versus no major bleeding (blue). Lines represent medians; shaded areas, interquartile ranges. Numbers denote sample size at each time point. Sample sizes differ between panels and reflect the anticoagulation regimen used (heparin monitored by anti-Xa, argatroban by anti-IIa assay). Episodes with major bleeding showed lower heparin doses and anti-Xa activity, particularly in the post-exchange period, consistent with dose reduction in response to bleeding. Argatroban doses and levels remained largely comparable between groups. Both dosing and monitoring parameters showed considerable day-to-day variability.

**Figure S8. Daily minor bleeding rates across the 15-day observation period centered on ECMO circuit exchange.**


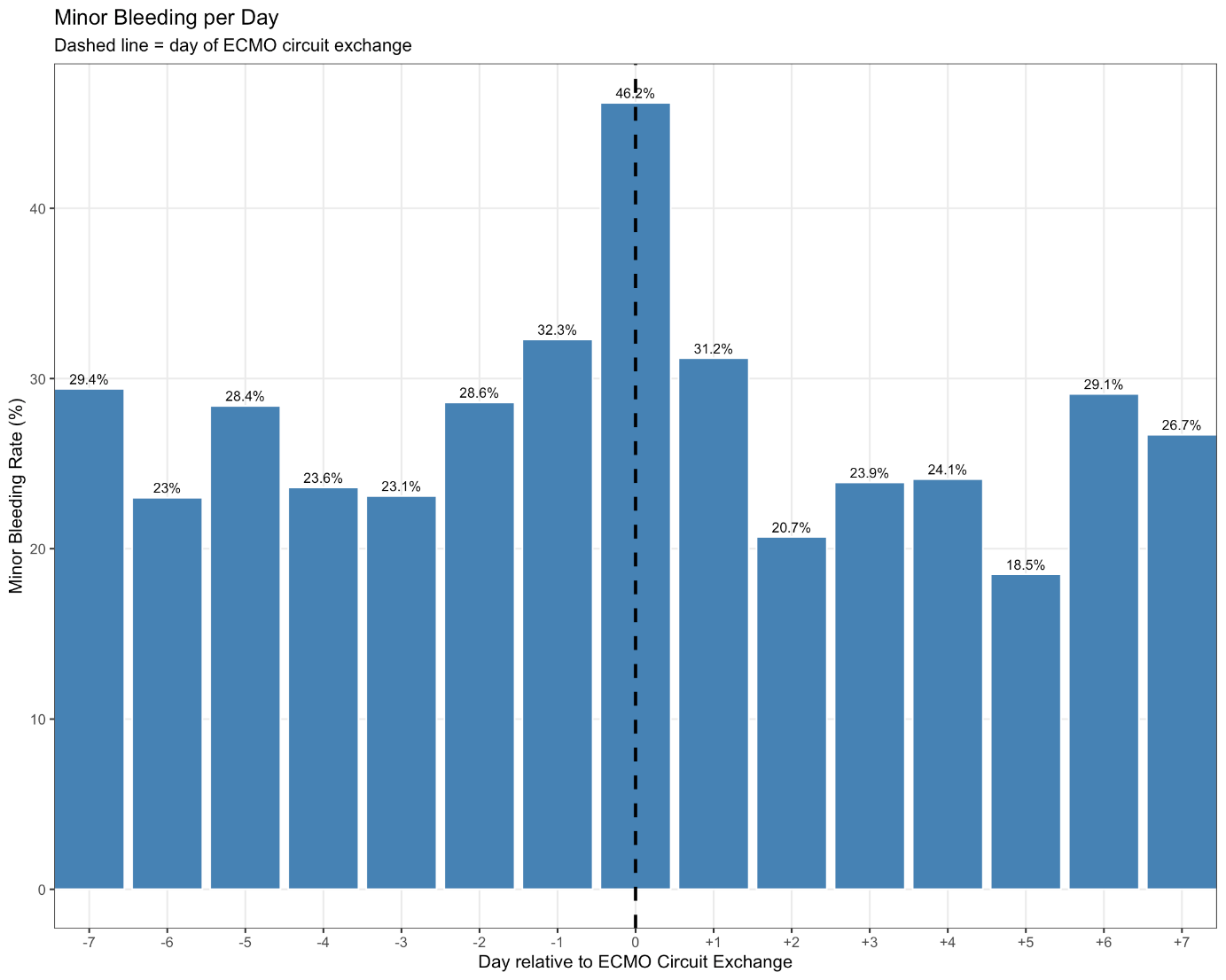


Bars represent the proportion of episodes with minor bleeding on each day relative to circuit exchange (day 0, dashed line). Minor bleeding peaked on the day of circuit exchange (46.2%) and remained frequent throughout the observation period (range 18.5–32.3% on remaining days), without the sustained post-exchange decline observed for major bleeding.

**Tables S4A-G. Linear mixed model results for longitudinal trajectories of laboratory parameters surrounding ECMO circuit exchange.**

| **A) Fibrinogen (g/L)** | Estimate | SE | 95% CI | p value |
| --- | --- | --- | --- | --- |
| Baseline value | 4.57 | 0.27 | 4.04 to 5.10 | <0.001 |
| Pre-exchange trend | -0.51 | 0.07 | -0.65 to -0.37 | <0.001 |
| Acceleration of change | -0.06 | 0.01 | -0.07 to -0.04 | <0.001 |
| **Immediate post-exchange shift** | -0.29 | 0.16 | -0.60 to 0.03 | 0.071 |
| Change in trajectory post-exchange | 0.97 | 0.14 | 0.68 to 1.25 | <0.001 |
| Bleeding-associated change | -0.92 | 0.18 | -1.27 to -0.58 | <0.001 |
| Cumulative circuit exchange effect | -0.32 | 0.06 | -0.43 to -0.21 | <0.001 |

| **B) D-dimer (mg/L)** | Estimate | SE | 95% CI | p value |
| --- | --- | --- | --- | --- |
| Baseline value | 24.77 | 1.60 | 21.64 to 27.91 | <0.001 |
| Pre-exchange trend | 3.97 | 0.48 | 3.02 to 4.91 | <0.001 |
| Acceleration of change | 0.38 | 0.07 | 0.25 to 0.51 | <0.001 |
| **Immediate post-exchange shift** | -11.61 | 1.13 | --13.82 to -9.40 | 0.071 |
| Change in trajectory post-exchange | -5.75 | 1.00 | -7.72 to -3.78 | <0.001 |
| Bleeding-associated change | 3.92 | 1.22 | 1.53 to 6.30 | 0.001 |
| Cumulative circuit exchange effect | --0.11 | 0.34 | -0.78 to 0.56 | 0.749 |

| **C) Platelet count (x10^9^)** | Estimate | SE | 95% CI | p value |
| --- | --- | --- | --- | --- |
| Baseline value | 122.51 | 7.71 | 107.38 to 137.63 | <0.001 |
| Pre-exchange trend | -7.15 | 2.27 | -11.60 to -2.69 | 0.002 |
| Acceleration of change | -0.38 | 0.31 | -0.98 to 0.22 | 0.217 |
| **Immediate post-exchange shift** | -6.67 | 5.27 | --17.02 to 3.67 | 0.206 |
| Change in trajectory post-exchange | 11.57 | 4.71 | 2.33 to 20.81 | 0.014 |
| Bleeding-associated change | -19.83 | 5.69 | -31.00 to -8.67 | <0.001 |
| Cumulative circuit exchange effect | --1.98 | 1.59 | -5.10 to 1.14 | 0.214 |

| **D) INR** | Estimate | SE | 95% CI | p value |
| --- | --- | --- | --- | --- |
| Baseline value | 1.19 | 0.04 | 1.12 to 1.27 | <0.001 |
| Pre-exchange trend | 0.04 | 0.01 | 0.02 to 0.06 | <0.001 |
| Acceleration of change | 0.01 | 0.00 | 0.00 to 0.01 | <0.001 |
| **Immediate post-exchange shift** | -0.00 | 0.02 | -0.05 to 0.04 | 0.878 |
| Change in trajectory post-exchange | -0.07 | 0.02 | -0.11 to -0.04 | <0.001 |
| Bleeding-associated change | 0.09 | 0.02 | 0.05 to 0.14 | <0.001 |
| Cumulative circuit exchange effect | 0.03 | 0.01 | 0.02 to 0.04 | <0.001 |

| **E) aPTT (s)** | Estimate | SE | 95% CI | p value |
| --- | --- | --- | --- | --- |
| Baseline value | 55.06 | 1.65 | 51.83 to 58.30 | <0.001 |
| Pre-exchange trend | +1.14 | 0.59 | −0.02 to 2.29 | 0.054 |
| Acceleration of change | +0.11 | 0.08 | −0.05 to 0.26 | 0.179 |
| **Immediate post-exchange shift** | −3.77 | 1.36 | −6.44 to −1.09 | 0.006 |
| Change in trajectory post-exchange | −1.44 | 1.22 | −3.83 to 0.95 | 0.238 |
| Major Bleeding-associated change | +2.61 | 1.45 | −0.24 to 5.46 | 0.073 |
| Cumulative circuit exchange effect | +1.24 | 0.32 | 0.60 to 1.88 | <0.001 |

| **F) Hemoglobin (g/dL)** | Estimate | SE | 95% CI | p value |
| --- | --- | --- | --- | --- |
| Baseline value | 9.09 | 0.15 | 8.79 to 9.39 | <0.001 |
| Pre-exchange trend | −0.22 | 0.04 | −0.31 to −0.14 | <0.001 |
| Acceleration of change | −0.02 | 0.01 | −0.04 to −0.01 | <0.001 |
| **Immediate post-exchange shift** | −0.03 | 0.10 | −0.23 to 0.17 | 0.743 |
| Change in trajectory post-exchange | +0.40 | 0.09 | 0.22 to 0.58 | <0.001 |
| Major Bleeding-associated change | −0.81 | 0.11 | −1.02 to −0.59 | <0.001 |
| Cumulative circuit exchange effect | −0.03 | 0.03 | −0.09 to 0.02 | 0.184 |

| **G) Free Hemoglobin (mg/L)** | Estimate | SE | 95% CI | p value |
| --- | --- | --- | --- | --- |
| Baseline value | 132.73 | 19.60 | 94.28 to 171.18 | <0.001 |
| Pre-exchange trend | +18.69 | 8.04 | 2.91 to 34.47 | 0.020 |
| Acceleration of change | +1.92 | 1.09 | −0.21 to 4.05 | 0.077 |
| **Immediate post-exchange shift** | −10.80 | 18.71 | −47.51 to 25.91 | 0.564 |
| Change in trajectory post-exchange | −33.22 | 16.68 | −65.94 to −0.50 | 0.047 |
| Major Bleeding-associated change | +43.12 | 22.12 | −0.41 to 86.66 | 0.052 |
| Cumulative circuit exchange effect | −6.35 | 3.60 | −13.41 to 0.71 | 0.078 |

Models included nested random intercepts for episodes within patients. Time was centered at day 0 (exchange day), with days -7 to 0 representing the pre-exchange phase and days +1 to +7 representing the post-exchange phase. Baseline value: Expected value at the exchange day (day 0), pre-exchange phase, in absence of major bleeding, at first exchange Pre-exchange trend: Daily change during the pre-exchange phase; Acceleration of change: Quadratic time effect indicating non-linear trajectory (positive = accelerating increase or decelerating decrease; negative = accelerating decrease or decelerating increase); Immediate post-exchange shift: Instantaneous change in level following circuit exchange; Change in trajectory post-exchange: Difference in daily slope between post- and pre-exchange phases (interaction term); Post-exchange trend: Calculated daily change during the post-exchange phase (pre-exchange trend + change in trajectory); Major Bleeding-associated change: Difference in parameter level associated with major bleeding complications; Cumulative circuit exchange effect: Change in baseline level per additional ECMO circuit exchange. *Abbreviations: CI, confidence interval; ECMO, extracorporeal membrane oxygenation; fHb, free hemoglobin; Hb, hemoglobin; INR, international normalized ratio; aPTT, activated partial thromboplastin time; SE, standard error.*

**Table S5. Random effects variance components from linear mixed models.**

| **Parameter** | **Patient-Level SD** | **Episode-Level SD** | **Residual SD** | **ICC (Patient)** | **ICC (Total)** |
| --- | --- | --- | --- | --- | --- |
| Fibrinogen, g/L | 1.08 | 0.87 | 1.33 | 0.32 | 0.52 |
| D-dimer, mg/L | 5.73 | 5.19 | 9.47 | 0.22 | 0.40 |
| Platelet count, x10^9^/L | 28.15 | 24.05 | 45.04 | 0.23 | 0.40 |
| INR | 0.17 | 0.10 | 0.17 | 0.41 | 0.56 |
| aPTT, s | 5.43 | 4.52 | 11.65 | 0.16 | 0.27 |
| Hemoglobin, g/dL | 0.67 | 0.36 | 0.86 | 0.34 | 0.44 |
| Free Hemoglobin, mg/L | 55.52 | 45.05 | 154.08 | 0.11 | 0.18 |

*SD = standard deviation of random effect (square root of variance component), Patient-Level SD = between-patient variability; Episode-Level SD = between-episode variability within patients; Residual SD = within-episode (day-to-day) variability, ICC_Patient_ = Var_Patient_ / (Var_Patient_ + Var_Episode_ + Var_Residual_); proportion of variance attributable to patient-level differences, ICC_Total_ = (Var_Patient_ + Var_Episode_ ) / Total Variance; proportion of variance explained by the nested random effects structure, Models included nested random intercepts: (1|Patient_ID/ID).* *Abbreviations: ICC, intraclass correlation coefficient; INR, international normalized ratio; aPTT, activated partial thromboplastin time; SD, standard deviation.*

**Table S6. Independent risk factors for major bleeding during ECMO support.**

| **Predictor** | **OR (95% CI)** | **p-value** |
| --- | --- | --- |
| Post-exchange phase | 0.32 (0.10 to 0.98) | 0.046 |
| Fibrinogen | 0.60 (0.37 to 0.96) | 0.035 |
| Platelet count | 0.58 (0.38 to 0.90) | 0.014 |
| D-dimer | 1.39 (0.96 to 2.00) | 0.079 |
| INR | 1.35 (0.92 to 1.98) | 0.126 |
| Free Hemoglobin | 1.12 (0.81 to 1.53) | 0.493 |
| WBC | 2.00 (1.43 to 2.82) | <0.001 |

Odds ratios (95% confidence intervals) derived from multivariable generalized linear mixed models with nested random intercepts for episodes within patients. Laboratory predictors are z-standardized; odds ratios represent the change in bleeding risk per 1 standard deviation increase. *Abbreviations: OR, odds ratio; CI, confidence interval; INR, international normalized ratio; ECMO, extracorporeal membrane oxygenation; WBC, white blood cell count.*

**Table S7. Fraction of missing information (FMI) for multivariable model parameters.**

| **Predictor** | **FMI** | **Lambda** | **RIV** |
| --- | --- | --- | --- |
| Post-exchange phase | 0.033 | 0.031 | 0.032 |
| Fibrinogen | 0.087 | 0.085 | 0.093 |
| Platelet count | 0.043 | 0.042 | 0.044 |
| D-dimer | 0.055 | 0.053 | 0.056 |
| INR | 0.160 | 0.157 | 0.187 |
| Free Hemoglobin | 0.159 | 0.156 | 0.185 |
| WBC | 0.060 | 0.058 | 0.062 |
| Post-oxygenator PO_2_ | 0.164 | 0.161 | 0.192 |

FMI = fraction of missing information; proportion of total variance attributable to missing data. Lambda = proportion of variation between imputations relative to total variation. RIV = relative increase in variance due to nonresponse. FMI < 0.1 indicates minimal impact; 0.1–0.3 moderate impact; > 0.3 substantial impact of imputation. *Further abbreviations: INR, international normalized ratio; PO_2_, partial pressure of oxygen; WBC, white blood cell count.*

**Table S8. Time-lagged predictors of major bleeding during ECMO support.**

| **Predictor** | **Lag (days)** | **OR** | **95% CI** | **p** | **P_FDR_** |
| --- | --- | --- | --- | --- | --- |
| **Fibrinogen** | 1 | 0.45 | 0.30 to 0.68 | <0.001 | 0.002 |
|  | 2 | 0.66 | 0.44 to 0.97 | 0.036 | 0.164 |
|  | 3 | 1.10 | 0.71 to 1.69 | 0.679 | 0.827 |
|  | 4 | 1.22 | 0.77 to 1.94 | 0.397 | 0.695 |
|  | 5 | 0.92 | 0.57 to 1.49 | 0.731 | 0.834 |
|  | 6 | 0.75 | 0.44 to 1.28 | 0.294 | 0.658 |
|  | 7 | 0.89 | 0.48 to 1.65 | 0.700 | 0.827 |
| **Platelet count** | 1 | 0.77 | 0.55 to 1.09 | 0.139 | 0.387 |
|  | 2 | 0.88 | 0.63 to 1.23 | 0.454 | 0.726 |
|  | 3 | 1.11 | 0.80 to 1.54 | 0.531 | 0.762 |
|  | 4 | 1.00 | 0.71 to 1.40 | 0.986 | 0.986 |
|  | 5 | 1.15 | 0.81 to 1.63 | 0.425 | 0.707 |
|  | 6 | 1.13 | 0.78 to 1.64 | 0.520 | 0.762 |
|  | 7 | 1.01 | 0.65 to 1.57 | 0.965 | 0.983 |
| **D-dimer** | 1 | 2.11 | 1.48 to 3.01 | <0.001 | 0.002 |
|  | 2 | 1.69 | 1.19 to 2.39 | 0.004 | 0.032 |
|  | 3 | 1.18 | 0.82 to 1.69 | 0.364 | 0.658 |
|  | 4 | 0.99 | 0.69 to 1.41 | 0.951 | 0.983 |
|  | 5 | 1.05 | 0.72 to 1.53 | 0.799 | 0.844 |
|  | 6 | 1.22 | 0.80 to 1.85 | 0.357 | 0.658 |
|  | 7 | 1.33 | 0.78 to 2.25 | 0.293 | 0.658 |
| **INR** | 1 | 1.35 | 1.01 to 1.80 | 0.043 | 0.164 |
|  | 2 | 1.33 | 0.96 to 1.85 | 0.090 | 0.265 |
|  | 3 | 0.59 | 0.35 to 0.99 | 0.044 | 0.164 |
|  | 4 | 0.49 | 0.29 to 0.84 | 0.009 | 0.056 |
|  | 5 | 0.69 | 0.42 to 1.14 | 0.145 | 0.387 |
|  | 6 | 0.93 | 0.57 to 1.51 | 0.774 | 0.834 |
|  | 7 | 0.85 | 0.49 to 1.48 | 0.576 | 0.787 |
| **aPTT** | 1 | 1.44 | 1.12 to 1.85 | 0.005 | 0.035 |
|  | 2 | 1.45 | 1.12 to 1.88 | 0.004 | 0.032 |
|  | 3 | 1.08 | 0.84 to 1.40 | 0.557 | 0.780 |
|  | 4 | 0.95 | 0.73 to 1.24 | 0.692 | 0.827 |
|  | 5 | 0.96 | 0.73 to 1.26 | 0.758 | 0.834 |
|  | 6 | 1.05 | 0.80 to 1.38 | 0.709 | 0.827 |
|  | 7 | 1.07 | 0.78 to 1.48 | 0.678 | 0.827 |
| **Post-oxy PO_2_** | 1 | 0.71 | 0.55 to 0.92 | 0.010 | 0.056 |
|  | 2 | 0.76 | 0.57 to 1.02 | 0.067 | 0.221 |
|  | 3 | 1.05 | 0.75 to 1.48 | 0.763 | 0.834 |
|  | 4 | 1.13 | 0.81 to 1.58 | 0.467 | 0.726 |
|  | 5 | 1.54 | 1.00 to 2.36 | 0.049 | 0.172 |
|  | 6 | 1.18 | 0.78 to 1.77 | 0.429 | 0.707 |
|  | 7 | 0.89 | 0.56 to 1.41 | 0.623 | 0.827 |
| **fHb** | 1 | 1.19 | 0.87 to 1.62 | 0.269 | 0.655 |
|  | 2 | 1.18 | 0.84 to 1.66 | 0.337 | 0.658 |
|  | 3 | 0.81 | 0.51 to 1.28 | 0.363 | 0.658 |
|  | 4 | 0.42 | 0.18 to 0.97 | 0.042 | 0.164 |
|  | 5 | 0.76 | 0.43 to 1.33 | 0.334 | 0.658 |
|  | 6 | 1.47 | 0.96 to 2.23 | 0.074 | 0.230 |
|  | 7 | 1.32 | 0.84 to 2.10 | 0.229 | 0.583 |
| **Predictor** | **Lag (days)** | **OR** | **95% CI** | **p** | **P_FDR_** |
| **WBC** | 1 | 1.93 | 1.42 to 2.61 | <0.001 | 0.001 |
|  | 2 | 1.59 | 1.16 to 2.18 | 0.004 | 0.032 |
|  | 3 | 1.84 | 1.30 to 2.60 | <0.001 | 0.008 |
|  | 4 | 1.45 | 1.02 to 2.08 | 0.041 | 0.164 |
|  | 5 | 1.20 | 0.81 to 1.78 | 0.357 | 0.658 |
|  | 6 | 1.09 | 0.71 to 1.68 | 0.681 | 0.827 |
|  | 7 | 0.80 | 0.42 to 1.51 | 0.491 | 0.743 |

Generalized linear mixed models with binomial distribution and nested random intercepts for episodes within patients. Laboratory values were measured 1–7 days prior to daily outcome assessment. Predictors are z-standardized; odds ratios represent the change in bleeding risk per 1 standard deviation increase. P-values were adjusted for multiple comparisons (56 tests) using Benjamini-Hochberg false discovery rate (p_FDR_) correction. *Abbreviations: CI, confidence interval; ECMO, extracorporeal membrane oxygenation; INR, international normalized ratio; OR, odds ratio; PO_2_, partial pressure of oxygen; aPTT, partial thromboplastin time, WBC, white blood cell count.*

**Table S9. Phase-stratified predictors of major bleeding during ECMO support.**

| **Predictor** | **Pre-Exchange** | **Post-Exchange** |
| --- | --- | --- |
| **Fibrinogen** | 0.61 (0.31 to 1.21), p = 0.160 | 0.41 (0.17 to 0.99), p = 0.047 |
| **Platelet count** | 0.76 (0.46 to 1.24), p = 0.269 | 0.30 (0.12 to 0.74), p = 0.009 |
| **D-dimer** | 1.39 (0.81 to 2.38), p = 0.237 | 0.95 (0.49 to 1.84), p = 0.880 |
| **INR** | 1.24 (0.76 to 2.02), p = 0.399 | 1.54 (0.80 to 2.94), p = 0.194 |
| **Free Hemoglobin** | 0.95 (0.62 to 1.45), p = 0.809 | 1.15 (0.63 to 2.12), p = 0.649 |
| **WBC** | 2.56 (1.64 to 4.01), p < 0.001 | 2.28 (1.15 to 4.53), p = 0.019 |
| **Post-oxygenator PO_2_** | 0.86 (0.59 to 1.26), p = 0.440 | 1.15 (0.63 to 2.12), p = 0.648 |

Phase-stratified predictors of major bleeding before and after ECMO circuit exchange. Odds ratios (per 1 SD increase) from generalized linear mixed models with nested random intercepts for episodes within patients, fitted separately for the pre-exchange (days -7 to -1) and post-exchange (day +1 to +7) periods and pooled across 30 multiply imputed datasets using Rubin's rules. All models adjusted for time using natural cubic splines.

**Table S10. Time-lagged predictors of major bleeding (multivariable GLMMs)**

| **Predictor** | **Lag 1 day** | **Lag 2 day** | **Lag 3 day** | **Lag 4 day** | **Lag 5 day** | **Lag 6 day** | **Lag 7 day** |
| --- | --- | --- | --- | --- | --- | --- | --- |
| **D-dimer** | 1.70 (1.17–2.46) | 1.48 (1.03–2.13) | 1.16 (0.80–1.70) | 1.02 (0.70–1.49) | 1.05 (0.70–1.57) | 1.19 (0.76–1.86) | 1.40 (0.78–2.53) |
|  | *p = 0.005* | *p = 0.034* | *p = 0.429* | *p = 0.924* | *p = 0.829* | *p = 0.458* | *p = 0.259* |
| **Fibrinogen** | 0.62 (0.39–0.97) | 0.80 (0.52–1.22) | 1.17 (0.74–1.85) | 1.28 (0.79–2.09) | 0.88 (0.52–1.49) | 0.73 (0.41–1.31) | 0.91 (0.45–1.87) |
|  | *p = 0.035* | *p = 0.298* | *p = 0.507* | *p = 0.319* | *p = 0.637* | *p = 0.296* | *p = 0.802* |
| **Platelet count** | 0.90 (0.61–1.32) | 0.94 (0.65–1.36) | 1.02 (0.72–1.46) | 0.92 (0.64–1.32) | 1.18 (0.82–1.71) | 1.26 (0.84–1.89) | 1.13 (0.68–1.89) |
|  | *p = 0.599* | *p = 0.759* | *p = 0.901* | *p = 0.643* | *p = 0.371* | *p = 0.259* | *p = 0.628* |
| **WBC** | 1.81 (1.30–2.50) | 1.52 (1.09–2.12) | 1.83 (1.28–2.61) | 1.48 (1.03–2.13) | 1.18 (0.79–1.75) | 1.05 (0.67–1.64) | 0.75 (0.38–1.47) |
|  | *p = <0.001* | *p = 0.015* | *p = <0.001* | *p = 0.035* | *p = 0.424* | *p = 0.835* | *p = 0.402* |

| Odds ratios (95% confidence intervals) from separate multivariable generalized linear mixed models with nested random intercepts  for episodes within patients, using laboratory values measured 1–7 days before outcome assessment. All models adjusted for time  and intervention phase. Laboratory predictors are z-standardized; odds ratios represent the change in bleeding risk per 1 standard  deviation increase. *Abbreviations: CI, confidence interval; GLMM, generalized linear mixed model; OR, odds ratio; WBC, white blood  cell count.* |
| --- |

**Additional exploratory analyses**

**Figure S9. Laboratory parameter trajectories in patients with D-dimer >35 mg/L without ECMO circuit exchange.**


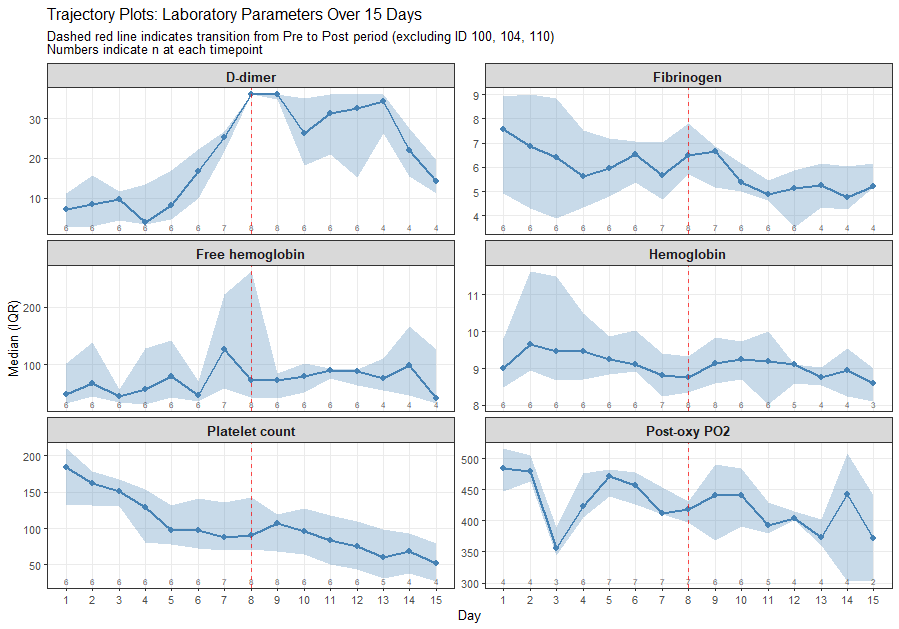


Data are presented as median (interquartile range). The dashed red line marks the first day of D-dimer peak (>35 mg/L). Data were censored after death (n=2) or ECMO explantation (n=3). Numbers below each panel indicate the number of patients with available data at each timepoint.

**Figure S10. Temporal distribution of major bleeding events and blood product transfusions in patients with D-dimer levels exceeding 35 mg/L without ECMO circuit exchange.**


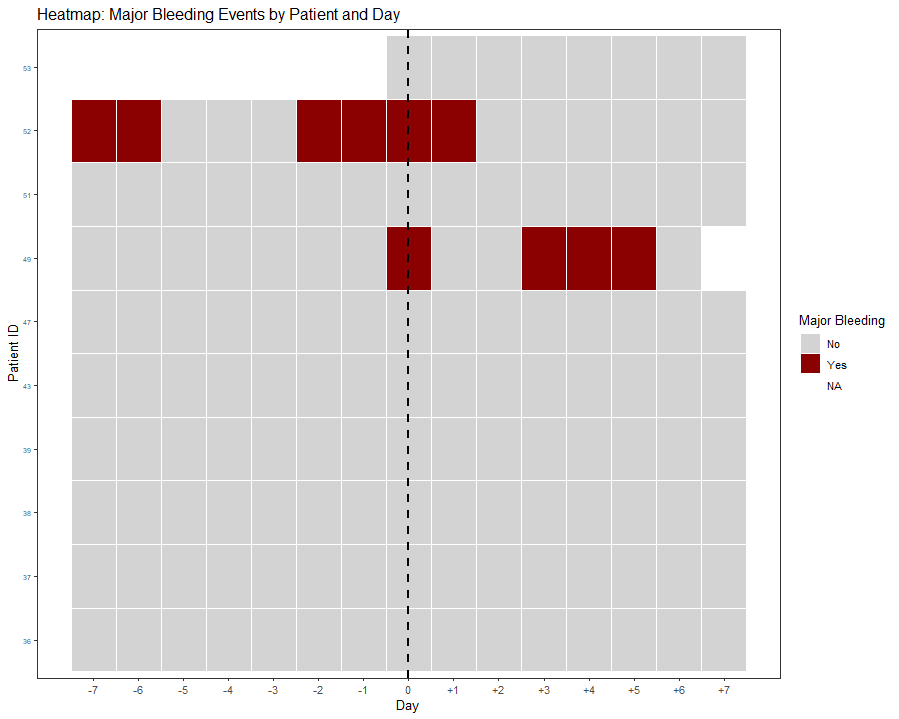

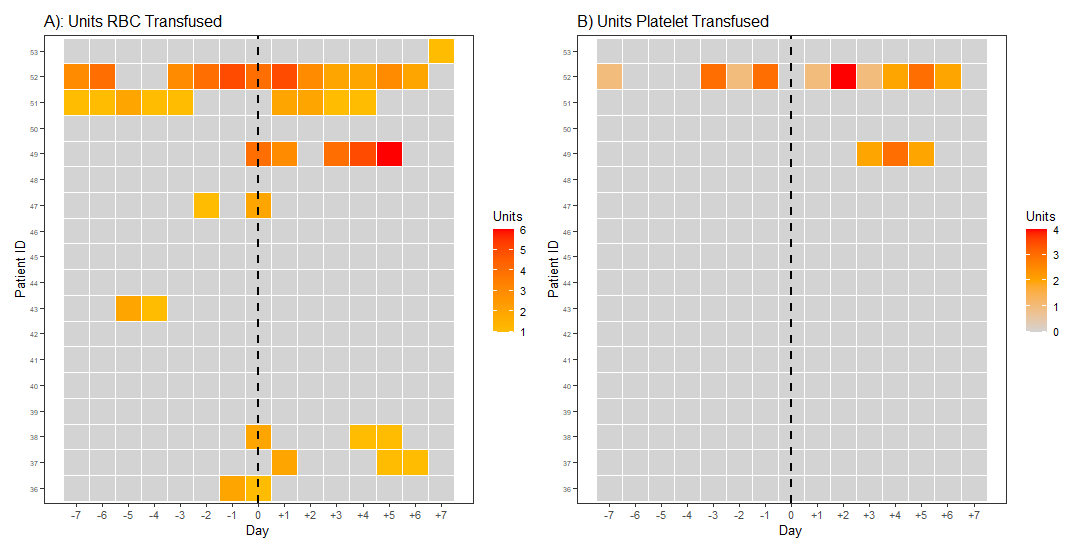


Each row represents an individual patient, and each column represents a day relative to the first observation of D-dimer > 35 mg/L (dashed vertical line). 1. **Dark red tiles indicate major bleeding events, gray tiles indicate no major bleeding,** and white tiles represent missing data. **2. (A)** Red blood cell (RBC) units transfused per day. **(B)** Platelet units transfused per day. Color intensity reflects the number of units transfused, ranging from yellow (low) to red (high). Gray tiles indicate days without transfusion (0 units).

**Figure S11. Laboratory parameter trajectories in the 7 days preceding death, stratified by cause of death.**


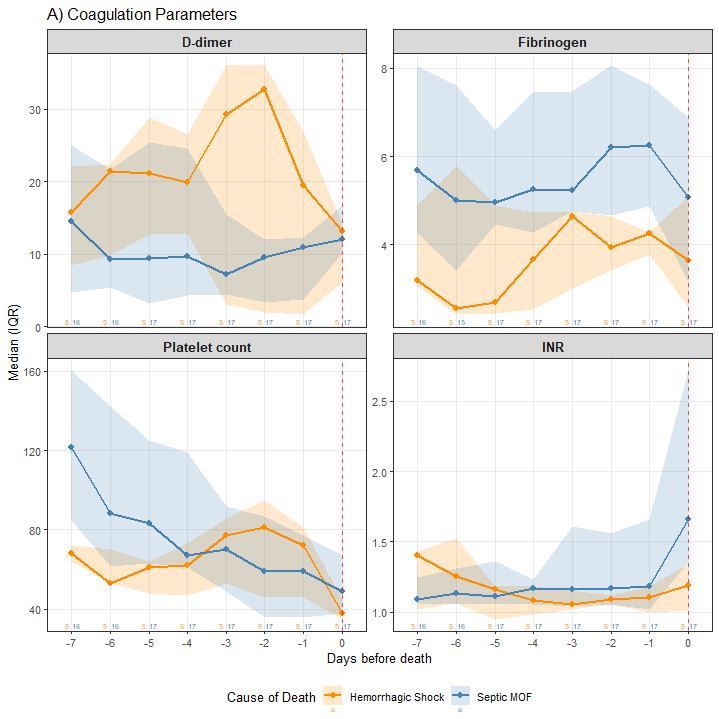

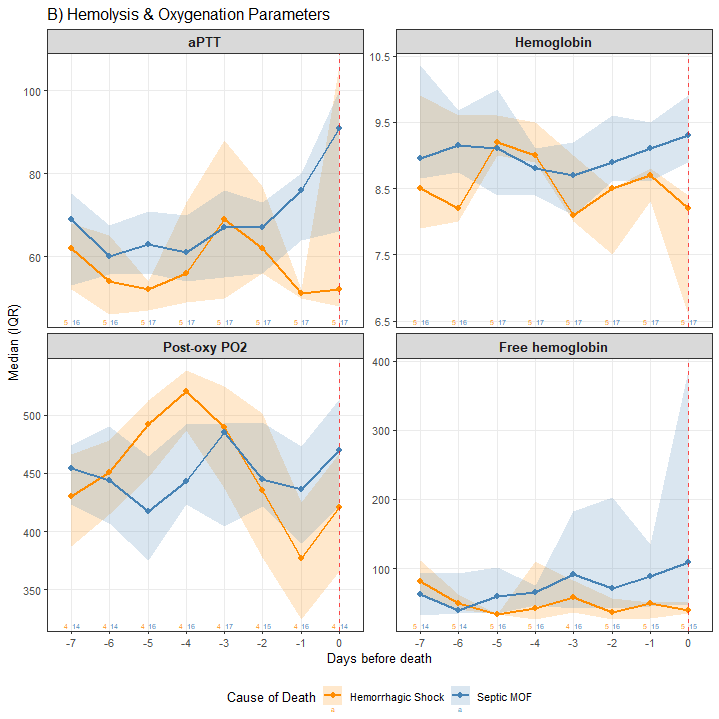


Data are presented as median (interquartile range) for patients who died during ECMO support (n=22), stratified by cause of death: septic multi-organ failure (blue, n=17) and hemorrhagic shock (orange, n=5). Day 0 represents the day of death. The dashed red line indicates the day of death. Laboratory trajectories in the 7 days preceding death differed between patients who died from septic multi-organ failure (n=17) and hemorrhagic shock (n=5). Patients with hemorrhagic shock exhibited persistently lower fibrinogen levels, more pronounced thrombocytopenia, and higher D-dimer levels throughout the observation period. In contrast, patients dying from septic multi-organ failure maintained relatively higher fibrinogen levels and showed a marked terminal rise in INR on the day of death. Due to the small sample size in the hemorrhagic shock group, formal statistical comparison was not performed.

**Figure S12. Temporal distribution of major bleeding events and blood product transfusions in deceased ECMO patients.**


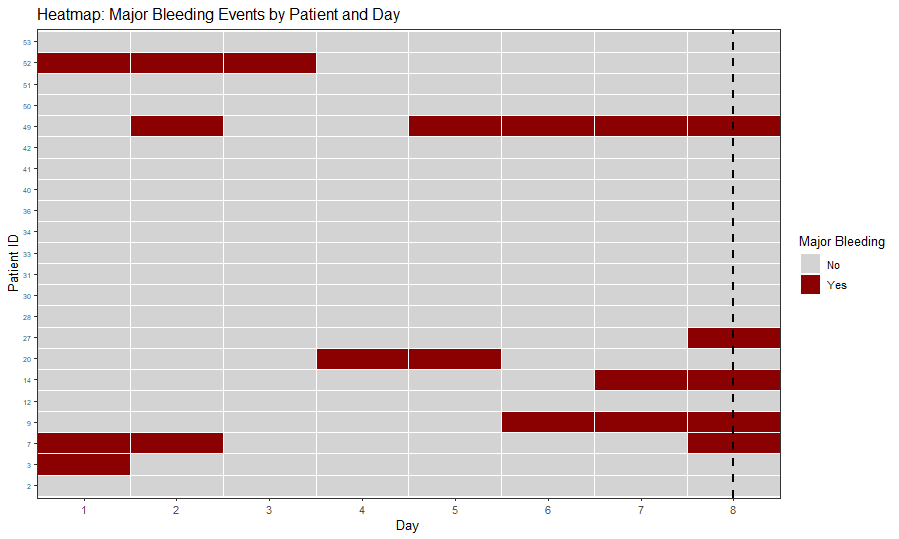


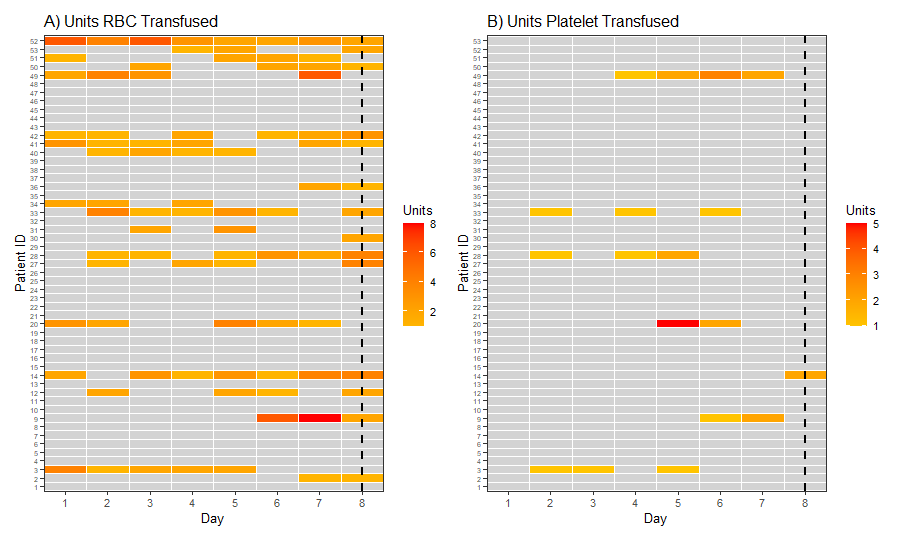


Each row represents an individual patient, and each column represents a day relative to death (Day 8 = day of death). 1.Dark red tiles indicate days with major bleeding events, gray tiles indicate days without major bleeding, and white tiles indicate missing data. 2. (A) Red blood cell (RBC) units transfused per day. (B) Platelet units transfused per day. Color intensity reflects the number of units transfused, ranging from yellow (low) to red (high). Gray tiles indicate days without transfusion. The dashed vertical line marks the day of death.
